# Variants Within *TSC2* Exons 25 and 31 Are Very Unlikely to Cause Clinically Diagnosable Tuberous Sclerosis

**DOI:** 10.1002/humu.22951

**Published:** 2016-01-12

**Authors:** Rosemary Ekong, Mark Nellist, Marianne Hoogeveen‐Westerveld, Marjolein Wentink, Jessica Panzer, Steven Sparagana, Warren Emmett, Natalie L. Dawson, Marie Claire Malinge, Rima Nabbout, Caterina Carbonara, Marco Barberis, Sergio Padovan, Marta Futema, Vincent Plagnol, Steve E. Humphries, Nicola Migone, Sue Povey

**Affiliations:** ^1^ Department of Genetics, Evolution and EnvironmentUniversity College LondonLondon WC1E 6BTUK; ^2^ Department of Clinical GeneticsErasmus MCRotterdam3015CNThe Netherlands; ^3^ Department of Pediatrics, Division of NeurologyThe Children's Hospital of PhiladelphiaPhiladelphiaPennsylvania19104‐4318; ^4^ Department of Neurology Perelman School of MedicineUniversity of PennsylvaniaPhiladelphiaPennsylvania19104; ^5^ Texas Scottish Rite Hospital for ChildrenDallasTexas75219; ^6^ University College London Genetics InstituteDarwin building, Gower StreetLondonWC1E 6BTUK; ^7^ Institute of Structural and Molecular BiologyUniversity College LondonLondonWC1E 6BTUK; ^8^ UF de Génétique Moléculaire, Département de Biochimie Génétique PBMMInstitut de Biologie en Santé CHU Angers49933 Angers, Cedex 9France; ^9^ Centre de Référence des Epilepsies RaresHôpital Universitaire Necker ‐ Enfants Malades75015ParisFrance; ^10^ Neonatology and Neonatal Intensive Care UnitS. Anna Hospital10126TorinoItaly; ^11^ Laboratory of Molecular GeneticsAzienda Ospedaliero Universitaria Città della Salute e della ScienzaPresidio OIRM S. Anna10126TorinoItaly; ^12^ CNR‐IBB UOS‐TO at MBCMolecular Biotechnology Center for University of Turin10126TorinoItaly; ^13^ Centre for Cardiovascular Genetics, British Heart Foundation LaboratoriesInstitute of Cardiovascular Sciences, University College LondonLondonUK; ^14^ Department of Medical SciencesUniversity of Turin10126TorinoItaly

**Keywords:** tuberous sclerosis, diagnosis, *TSC2*, alternative splicing, variants

## Abstract

Inactivating mutations in *TSC1* and *TSC2* cause tuberous sclerosis complex (TSC). The 2012 international consensus meeting on TSC diagnosis and management agreed that the identification of a pathogenic *TSC1* or *TSC2* variant establishes a diagnosis of TSC, even in the absence of clinical signs. However, exons 25 and 31 of *TSC2* are subject to alternative splicing. No variants causing clinically diagnosed TSC have been reported in these exons, raising the possibility that such variants would not cause TSC. We present truncating and in‐frame variants in exons 25 and 31 in three individuals unlikely to fulfil TSC diagnostic criteria and examine the importance of these exons in TSC using different approaches. Amino acid conservation analysis suggests significantly less conservation in these exons compared with the majority of *TSC2* exons, and *TSC2* expression data demonstrates that the majority of *TSC2* transcripts lack exons 25 and/or 31 in many human adult tissues. In vitro assay of both exons shows that neither exon is essential for TSC complex function. Our evidence suggests that variants in *TSC2* exons 25 or 31 are very unlikely to cause classical TSC, although a role for these exons in tissue/stage specific development cannot be excluded.

## Introduction

Tuberous sclerosis complex (TSC) is an autosomal‐dominant disease caused by inactivating mutations in either *TSC1* (MIM #605284) or *TSC2* (MIM #191092). TSC disease severity is variable with signs and symptoms ranging from hypomelanotic macules, to epilepsy, intellectual disability, autism, and multiple hamartomas in kidney, brain, heart, and lung. In TSC, about 70% of cases are due to new mutations [Sampson et al., 1989; Osborne et al., 1991; Au et al., 2007] and this presents a challenge for molecular diagnostics especially when the variant identified is not obviously disease causing. For example, some missense changes in *TSC2* have been associated with TSC in patients diagnosed with definite TSC [Sancak et al., 2005; Hoogeveen‐Westerveld et al., 2011], as well as cases in which symptoms are less severe and TSC is more often likely to be familial [Khare et al., 2001; O'Connor et al., 2003; Mayer et al., 2004; Jansen et al., 2006; Wentink et al., 2012].

Although there are well‐defined clinical diagnostic criteria for TSC [Northrup et al., 2013], it can still be difficult to establish a clinical diagnosis of TSC, particularly in young patients who do not yet exhibit typical TSC lesions, but may have severe but nonspecific symptoms such as epilepsy, intellectual disability, and/or autism. In these cases, a molecular diagnosis can be helpful. Indeed, the identification of a clearly inactivating *TSC1* or *TSC2* mutation is considered sufficient evidence for a diagnosis of TSC, even in the absence of clinical signs [Northrup et al., 2013]. Increasingly, next‐generation sequencing (NGS) of *TSC1*, *TSC2*, and many other genes simultaneously (gene panels, whole‐genome [WGS] or whole‐exome [WES] sequencing) is being used in diagnostic settings. NGS is also applied to population genetics in large‐scale sequencing studies, such as the NHLBI GO Exome Sequencing Project with data in the Exome Variant Server (EVS) and the Exome Aggregation Consortium (ExAC). In these studies, TSC is not assessed and the available phenotype data are very limited. This reliance on molecular diagnosis makes it even more important that the interpretation of any apparently pathological variant is correct.

The main recognized challenge to the molecular diagnosis of TSC has been (and still is) the “Variants of Uncertain Significance” (VUS), many of these being missense and in‐frame indels. The *TSC1* and *TSC2* genes encode core components of the TSC protein complex that is a critical negative regulator of the mechanistic target of rapamycin (mTOR) complex 1 (TORC1) [Dibble and Manning, 2013]. In vitro assays to determine the effects of *TSC1* and *TSC2* missense and in‐frame indels on TORC1 activity have proven useful in the ascertainment of the pathogenicity of variants previously reported as VUS [Hoogeveen‐Westerveld et al., 2011, 2013; Dunlop et al., 2011]. In these tests, assay results are summarized as the effect of the variants on the “TSC complex function” by which we mean “the inhibition of TORC1 activity.” An additional option for evaluating a VUS is to perform conservation analysis of orthologous protein sequences in multiple species. Several computational algorithms (e.g., PolyPhen, SIFT) incorporate protein alignments from multiple species into the evaluation of the effects of amino acid substitutions on protein function [Ng and Henikoff, 2003; Adzhubei et al., 2013]. In our experience, these algorithms were not reliable enough to classify individual VUS with confidence [Hoogeveen‐Westerveld et al., 2011]. However, this approach may still be applicable where high‐quality alignments of multiple species are utilized. Conservation analysis has been utilized as a secondary line of evidence in the classification of VUS in the *BRCA1/2* genes [Eggington et al., 2014].

The *TSC1* and *TSC2* Leiden Open Variation Databases (TSC LOVD) were made publicly available in 2006 and the *TSC2* LOVD now displays 2,232 different variants (November 13, 2015), with a further 300 variants available on querying. Of these 2,532 different variants, about half are considered to be certainly or probably pathogenic. All 41 coding *TSC2* exons, except exons 25 and 31, include both obviously truncating variants and fully confirmed missense variants that cause tuberous sclerosis. These two exons (25 and 31) have been shown to undergo alternative splicing in many tissues [Xiao et al., 1995; Xu et al., 1995; Olsson et al., 1996], although the extent and clinical significance of this is unclear. So far, we are not aware of confirmed reports of any pathological variants in these two exons in TSC cases. We have questioned the meaning of this observation over the 10 years of curating the TSC LOVD. To investigate the implications of this finding, we conducted function tests to assay expression constructs that lacked sequences corresponding to whole *TSC2* exons, before the cases with truncating variants described below came to our attention.

As curators of the TSC LOVD, we are often asked to assist in classifying new *TSC1* and *TSC2* variants and we recently encountered a frameshift variant in *TSC2* exon 25 and a nonsense variant in *TSC2* exon 31, in individuals who had not been diagnosed with TSC and as described below (cases 1 and 2) were unlikely to have the disease. Finding these two stop codons triggered further investigations on the two exons and we gathered existing evidence that might help clarify the significance of exons 25 and 31 in TSC. During our investigations, we became aware of an individual with an in‐frame deletion in *TSC2* exon 31 (case 3 below). None of our three cases met the diagnostic criteria for TSC. Therefore, to gain insight into the significance of these variants in TSC, we reviewed all variants mapping to exons 25 and 31 in the *TSC2* LOVD (http://www.lovd.nl/TSC2), performed conservation analysis, reviewed *TSC2* mRNA expression data, and conducted in vitro functional analysis. We conclude that *TSC2* exons 25 and 31 are not required for the TSC complex‐dependent inhibition of TORC1 and that variants in these exons are unlikely to cause TSC.

## Materials and Methods

### *TSC2* Exon and Variant Nomenclature

*TSC2* (GenBank NG_005895.1 GI:125662814) consists of 42 exons, including a 5' noncoding exon [ftp://ftp.ebi.edu.au/pub/databases/lrgex/LRG_487.xml]. However, the TSC community usually number *TSC2* exons from the start of the protein coding sequence making 41 exons in total, with the 5' noncoding exon numbered 1a. This convention is used here.

Nucleotide numbering corresponds to the *TSC2* cDNA sequence (GenBank NM_000548.3, GI:116256351) with +1 as A of the ATG translation initiation codon in the reference sequence, and the initiation codon as codon 1.

### Patients and Variants

Case 1: A predicted nonsense variant in *TSC2* exon 25 (c.2859dup, p.K954Qfs*6) was identified in an epilepsy gene panel test performed on DNA from a 10‐year‐old patient with epilepsy, but no clinical or radiographic findings supporting a diagnosis of TSC. The variant was reported as “pathogenic.”

Case 2: We analyzed rare (frequency <0.005) *TSC1* and *TSC2* variants identified in 1,805 individuals who had their whole exome sequenced as part of the UK10K project [UK10K Consortium et al.,^2015^]. Study participants consented to be included as controls for diseases that were not directly under study. As part of the search for “incidental findings” that might cause serious inherited disease we received 67 rare variants to assess. These included a predicted nonsense variant in *TSC2* exon 31 (c.3837C>G, p.Y1279*). The only accessible information on this individual is that they were recruited for a diabetes study. This variant is reported in the UK10K publication [UK10K Consortium et al., 2015].

Case 3: We identified an African American individual with intractable neonatal onset epilepsy and profound developmental delay, and an in‐frame deletion in *TSC2* exon 31 (*TSC2* c.3846_3855delinsG, p.S1282_G1285delinsR). This individual also carries the *TSC2* c.4007C>T (p.S1336L, exon 33) variant that has a minor allele frequency of 0.137%–0.296% in the African population (rs148527903; http://evs.gs.washington.edu/EVS/ and http://exac.broadinstitute.org/). Both variants were reported as “possibly pathogenic.” The individual has two café au lait spots, normal renal imaging, normal ophthalmology examination, no hypopigmented macules, and no brain MRI findings characteristic of TSC (no tubers, subependymal nodules, or areas of cortical dysplasia). A possible cardiac rhabdomyoma was detected on an echocardiogram obtained after the genetic test result. The most recent MRI at 4 years 9 months still shows no stigmata of TSC. *TSC2* c.3846_3855delinsG was inherited from a parent (32 years old) who has also been seen at the TSC clinic. The parent has a single hypopigmented macule, but no obvious stigmata of TSC. Of note is a different *TSC2* exon 31 variant (*TSC2* c.3846_3854del) that is predicted to encode an identical protein (p.S1282_G1285delinsR). This variant was reported in a large‐scale WES study of individuals with autism spectrum disorder and epilepsy [Schaaf et al., 2011].

### Conservation Analysis and Variants in *TSC2* Exons 25 and 31

To assess the conservation of *TSC2* exons 25 and 31 and review the classification of variants within these two exons, a manual alignment of 163 orthologous vertebrate TSC2 protein sequences was constructed. For the comparison of conservation in exons 25 and 31 to other *TSC2* exons, we performed a simple mathematical analysis and a more comprehensive statistical analysis using Scorecons [Valdar, 2002] and Wilcoxon Rank‐Sum test [R Core Team, 2015].

The classification of all variants in *TSC2* exons 25 and 31 reported to us (http://www.lovd.nl/TSC2) were reviewed.

### Expression Analysis

Literature searches were performed and data in the NCBI (http://www.ncbi.nlm.nih.gov/gene/7249) and Genotype‐Tissue Expression (GTEx) databases (http://www.gtexportal.org/home/) were used for expression analyses. A multiple transcript sequence alignment was performed using Clustal Omega (http://www.ebi.ac.uk/Tools/msa/clustalo/).

### Functional Analysis

To assess the functional importance of exons 25 and 31, *TSC2* expression constructs with in‐frame deletions of sequences corresponding to specific exons were derived from the original full‐length *TSC2* expression construct containing exon 25 but lacking exon 31 [Nellist et al., 2005]. Site‐directed mutagenesis (SDM) was used to delete these sequences. Expression constructs lacking exons 3, 4, 5, 6, 9, 12, 19, 22, and 41 served as controls for the expression construct lacking exon 25. To derive a *TSC2* expression construct containing exon 31 (referred to as +ex31) an Eco47III fragment from a partial cDNA clone containing exon 31 was cloned into the original *TSC2* expression construct. The p.R611Q (exon 16), p.S1282_G1285delinsR (exon 31), and p.S1336L (exon 33) variants were derived from the +ex31 expression construct by SDM. For each construct, the entire *TSC2* open‐reading frame was checked by sequencing to ensure that no additional nucleotide changes were introduced by the SDM procedure. A standard functional assay [Hoogeveen‐Westerveld et al., 2011] was applied to determine the effects of the variants on TSC2 protein stability (estimated from the TSC2 protein signal), the TSC1‐TSC2 protein interaction (estimated from the TSC1 signal), and on TORC1 activity (estimated from the ratio of T389‐phosphorylated p70 S6 kinase (S6K): total S6K).

## Results

### Conservation Analysis and Pathogenicity of Variants in *TSC2* Exons 25 and 31

*TSC2* exons 25 and 31 are conserved in vertebrates down to the lamprey (Supp. Fig. S1, A and B). The *TSC2* LOVD contains 17 different variants (excluding contiguous exon deletions) mapping to *TSC2* exon 25, or to the adjacent intronic sequences; 14 of these (five intronic, two silent, and seven missense variants) are classified as not pathogenic (not causing TSC) and three missense variants (p.K954R, p.S960F, and p.E984Q) are unclassified. One of the three unclassified missense variants (p.K954R) occurs naturally in 24/161 vertebrate species analyzed, including 11/83 mammals, whereas codons 960 (22/161) and 984 (19/161) in the alignment have different substituted amino acids to those found in our database (Supp. Fig. S1A).

The *TSC2* LOVD lists 16 different variants (excluding contiguous exon deletions) mapping to exon 31, of which 15 (10 intronic, one silent, one in‐frame indel, and three missense variants) are classified as not pathogenic. One intronic variant remains unclassified. Amino acids corresponding to the three missense variants (p.S1276F, p.S1282G, and p.V1291I) occur naturally in one or more of the vertebrates analyzed (Supp. Fig. S1B).

Our simple mathematical analysis showed that exon 25 has an average degree of conservation, whereas exon 31 was among the least conserved. Comparisons of exons 25 and 31 to two other comparatively more conserved exons (7 and 22) are shown (Supp. Fig. S2). A more comprehensive conservation analysis showed agreement with the simple analysis described above. There was a wide range of conservation across the 41 *TSC2* exons and the general pattern was similar with or without fish sequences, apart from a few exceptions (Supp. Fig. S3A and B). The amino acids encoded by exon 31 were less conserved on average when compared with those encoded by exon 25 (Supp. Fig. S3A–D), and the statistical analysis showed that the distributions of conservation scores for exons 25 and 31 were significantly lower in value (*P* < 0.05; Wilcoxon Rank‐Sum test) as compared with the distribution of scores in most of the *TSC2* exons (Supp. Table S1).

### Expression Analysis

The first full‐length human *TSC2* cDNA reported was from human fetal brain and included exon 25, but not exon 31 [European Chromosome 16 Tuberous Sclerosis Consortium, 1993]. Exon 31 was subsequently reported in rat and confirmed in human cDNA [Xu et al., 1995; Kimura et al., 2006]. We reviewed publications that describe the tissue distribution of *TSC2* transcripts with and without exon 25 in rodents and in limited human material [Xiao et al., 1995; Xu et al., 1995; Olsson et al., 1996]. Although exon 25 and the first codon in exon 26 were often absent from *TSC2* transcripts in rodents and humans, most tissues expressed some transcripts containing these sequences [Xiao et al., 1995; Xu et al., 1995; Olsson et al., 1996]. *TSC2* transcripts lacking exon 25 (−ex25) were predominant in samples from adult human brain and adult human cerebellum [Xu et al., 1995], whereas human fetal muscle had more transcripts containing exon 25 (+ex25) [Xu et al., 1995]. A quantitative study [Olsson et al., 1996] estimated the ratio of +ex25:−ex25 in human fetal brain at 1:1; in three other fetal tissues, the −ex25 transcript was predominant. Human fetal kidney and fetal brain were found to have mainly the transcript lacking exon 31 [Xiao et al., 1995].

We searched two databases with *TSC2* transcript information: The Ensembl Genome Browser at http://www.ensembl.org/ and the National Center for Biotechnology Information Gene database at http://www.ncbi.nlm.nih.gov/gene/7249. Six of the 26 different *TSC2* transcripts listed in Ensembl were aligned using Clustal Omega to confirm the exact sequences that were absent from each transcript. The remaining 20 transcripts were not analyzed as these are too short to produce a functional protein. In the NCBI database (*Homo sapiens* annotation release 106), tracks of intron spanning *TSC2* RNA‐sequencing (RNA‐seq) reads generated from alignments of RNA‐seq data from the Bodymap2 dataset (http://www.ncbi.nlm.nih.gov/geo/query/acc.cgi?acc=GSE30611) of 16 adult tissue samples were analyzed. Each tissue in the Bodymap2 dataset is derived from a different single individual. To obtain evidence for tissue‐specific alternative splicing of *TSC2* exons 25 and 31, we examined tracks of intron spanning RNA‐seq reads for these exons in each Bodymap2 tissue sample. Analysis of the BodyMap2 tracks showed evidence for the expected sequence joining each pair of adjacent exons in the majority of reads, except those involving exons 25 and 31 where a minority of reads (∼6% and ∼11%, respectively) showed a join between adjacent exons, indicating that transcripts encoding exons 25 and/or 31 are relatively scarce. Exon 25 was just detectable in heart, lymph node, skeletal muscle, and thyroid tissue samples, but not detectable in adipose, adrenal, brain, breast, colon, kidney, liver, lung, ovary, prostate, testes, or white blood cells. Exon 31 was detectable in transcripts from adipose, adrenal, brain, breast, colon, kidney, lung, prostate, skeletal muscle, testes, thyroid, and white blood cells, but not in heart, liver, lymph node, or ovary.

A similar analysis was performed for 27 different tissues that are reported in a study of normal adult tissues taken from 95 different individuals [Fagerberg et al, 2014], and are added to the *TSC2* dataset at NCBI (*Homo sapiens* Annotation Release 107) [https://goo.gl/Gu0JWq]. Some of the tissues examined in the BodyMap2 dataset are not included in this study, for example, skeletal muscle, breast, and white blood cells. The same tissue type was analyzed from at least two individuals and exons 25/31 are also just detectable in some individuals and not evident at all in others. Aggregate reads show that the inclusion of exons 25 and 31 is 7.8% and ∼23% of transcripts, respectively, supporting the abundance of the −ex25 and −ex31 transcripts in adult tissues. Since the tracks in both studies are from short reads, it was not possible to determine whether the observation of both exons, for example, in skeletal muscle and thyroid tissue, indicated that both exons are present in the same transcript in those tissues. No other *TSC2* exons showed evidence for alternative splicing in these datasets at NCBI.

Analysis of a third RNA‐seq dataset from 175 individuals in the genotype‐tissue expression (GTEx) project [GTEx Consortium, 2015] also confirmed the relatively low abundance of *TSC2* transcripts containing exons 25 and/or 31 in 51 different tissues from adults (Supp. Fig. S4; Supp. Tables S2 and S3).

In all the three RNA‐seq datasets described above, both exons 25 and 31 are present in very low amounts in adult tissues and symptoms restricted to specific cell types cannot be excluded if there is some expression of abnormal +ex25 and/or +ex31 transcripts.

### Effects of *TSC2* Exon‐Specific Deletions and Variants on the Canonical TSC Complex Function

**Figure 1:**
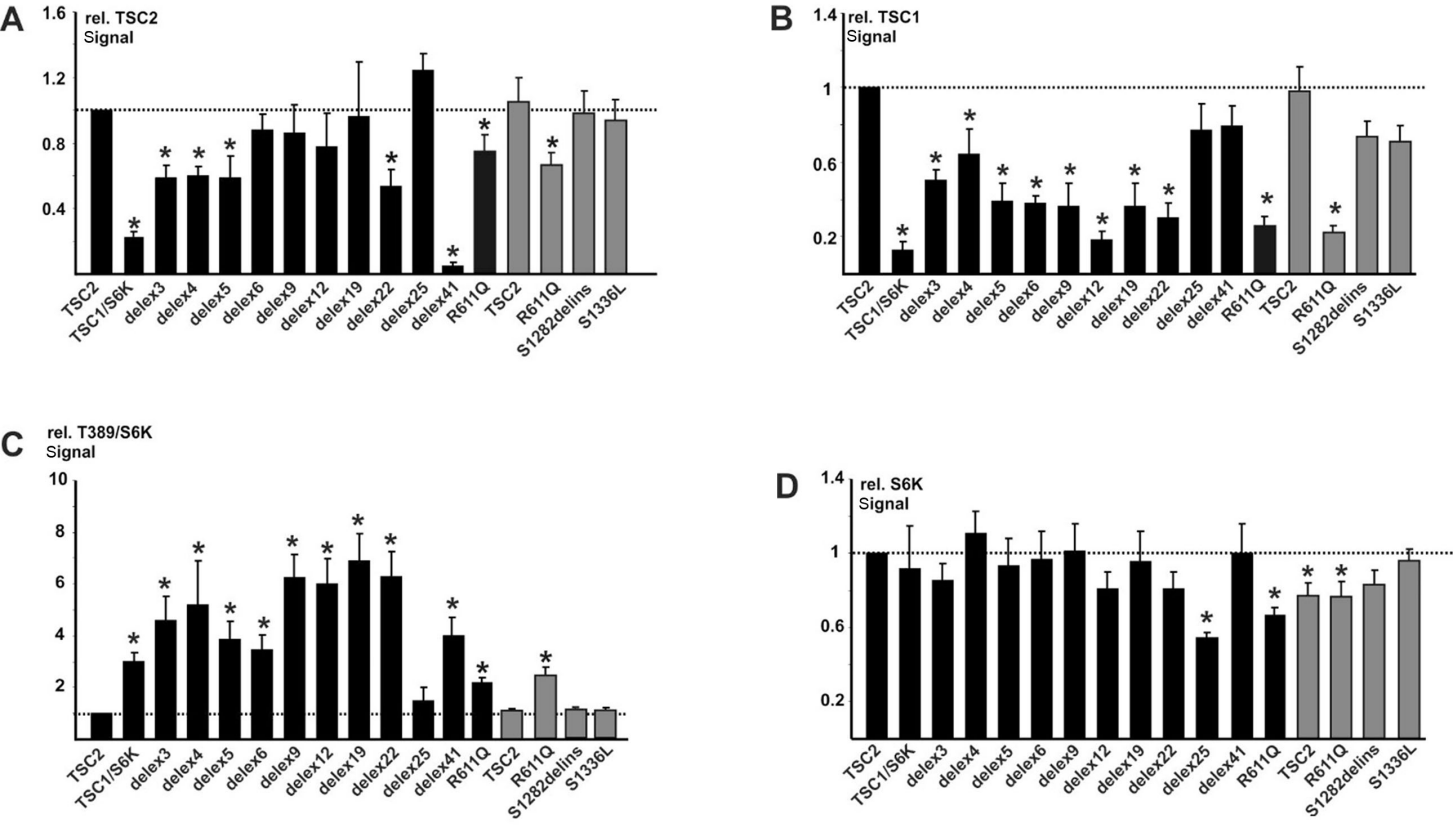
Functional assessment. *TSC2* expression constructs were derived from the original wild‐type construct containing exon 25 but lacking exon 31 (TSC2), indicated in black. Nonitalicized “TSC2” refers to the protein and is used as such throughout this legend. The pathogenic TSC2 p.R611Q (R611Q) control, and the p.S1282_G1285delinsR (S1282delins) and p.S1336L variants identified in case 3 are indicated. In‐frame exon‐specific deletion constructs are referred to as delex3 (for deletion of exon 3), delex4, and so forth. Expression constructs encoding exon 31 are indicated in gray. *TSC2* expression constructs were transfected into HEK 293T cells together with *TSC1* and *S6K* expression constructs. The signals for TSC2, TSC1, total S6K (S6K), and T389‐phosphorylated S6K (T389) were determined per variant, relative to the wild‐type control (TSC2) in at least three independent transfection experiments by immunoblotting, as described previously [Hoogeveen‐Westerveld et al., 2011]. Results for cells transfected with *TSC1* and *S6K* only (TSC1/S6K) are shown for comparison. The mean TSC2 (**A**), TSC1 (**B**), and S6K (**D**) signals and mean T389/S6K ratio (**C**) are shown. In each case, the dotted line indicates the signal or ratio corresponding to wild‐type TSC2 (+exon 25; −exon 31) (TSC2; = 1.0). Error bars represent the standard error of the mean; variants that showed significant differences from the wild‐type control (TSC2; +exon 25; −exon 31) are indicated with an asterisk (*P* < 0.05; Student's *t*‐test). **A**: Mean signals for the TSC2 variants, relative to wild‐type TSC2 (+exon 25; −exon 31). Note that the TSC2 signal is significantly reduced by deletion of exons 3, 4, 5, or 22, or by the pathogenic R611Q substitution. Deletion of sequences corresponding to exon 41 (delex41) removes the epitope recognized by the antibody used for TSC2 detection. **B**: Mean TSC1 signals in the presence of the TSC2 variants relative to wild‐type TSC2 (+exon 25; −exon 31). TSC1 signals were significantly reduced in the presence of the delex3, 4, 5, 6, 9, 12, 19, and 22 variants, and in the presence of the TSC2 R611Q substitution. **C**: Mean T389/S6K ratios in the presence of the TSC2 variants, relative to wild‐type TSC2 (+exon 25; −exon 31). The T389/S6K ratios were significantly increased in the presence of the R611Q, delex3, 4, 5, 6, 9, 12, 19, 22 and 41 variants, as well as in the absence of TSC2 (TSC1/S6K).**D**: Mean total S6K signals in the presence of the TSC2 variants, relative to wild‐type TSC2 (+exon 25; −exon 31).

To investigate the functional importance of exons 25 and 31 compared with other *TSC2* exons, we derived a full‐length *TSC2* expression construct containing exon 31 (referred to as +ex31) and utilized existing data from a series of *TSC2* expression constructs lacking exons 3, 4, 5, 6, 9, 12, 19, 22, 25, and 41. These exons have varying numbers of confirmed TSC‐causing variants (exon 3 [67 variants], exon 4 [50 variants], exon 5 [63 variants], exon 6 [22 variants], exon 9 [64 variants], exon 12 [48 variants], exon 19 [61 variants], exon 22 [35 variants], and exon 41 [18 variants]) and exon 25 has none. Previous work had shown that three missense variants mapping to exon 25 (p.R951S, p.R988P [Hoogeveen‐Westerveld et al., 2011] and p.N958S [Hoogeveen‐Westerveld et al., 2013]) did not affect TSC complex function. The TSC1–TSC2 protein interaction is important for maintaining the activity of the TSC complex, and therefore for effective inhibition of TORC1. Reductions in the TSC1 signals indicate that the TSC1–TSC2 protein interaction is disrupted [Hoogeveen‐Westerveld et al., 2011]. It was observed that deletion of exons corresponding to the TSC1 interaction domain of the TSC2 protein, or the introduction of a pathogenic amino acid substitution (TSC2 p.R611Q) in this domain, significantly reduced the TSC1 protein signal (Fig. 1B) and prevented the TSC complex‐dependent inhibition of TORC1 activity, as assessed by the T389 phosphorylation status of a S6K reporter (Fig. 1C). In contrast, deletion of *TSC2* exon 25 (delex25) or inclusion of exon 31 (+ex31) did not significantly affect TSC1 protein signals (Fig. 1B) or S6K‐T389 phosphorylation (Fig. 1C), compared with the original wild‐type TSC2 protein. Also, the presence of sequences corresponding to exon 31 did not rescue the activity of the p.R611Q variant. Therefore, we did not obtain evidence for a clear functional effect of exons 25 or 31 on TSC complex activity in our assay. Similarly, we did not obtain evidence that either the p.S1282_G1285delinsR (S1282delins) or p.S1336L variant affected TSC2 protein stability, TSC1–TSC2 protein interaction, or TSC complex‐dependent inhibition of TORC1 activity (Fig. 1A–C). Deletion of exon 25 (delex25) did result in a nonsignificant reduction in TSC1 protein signals, a nonsignificant increase in S6K‐T389 phosphorylation and a significant decrease in the total S6K signal. The low S6K signal suggests that the transfection efficiency for the delex25 variant was reduced compared with the other variants tested, and it is possible that the deletion of exon 25 sequences might have a subtle effect on TSC complex activity. Nonetheless, compared with the pathogenic R611Q variant, and the negative control transfection (TSC1/S6K only), the delex25 variant was still able to stabilize the TSC1 protein and inhibit TORC1.

## Discussion

Clinical diagnosis of TSC can be problematic due to phenotypic variability, even between affected individuals from the same family who have the same TSC‐causing variant. Therefore, adding genetic criteria [Northrup et al., 2013] should make TSC diagnosis less ambiguous. However, the increasing application of NGS‐based gene panels, WES and WGS in diagnostic settings, as well as the increasing amount of data from large‐scale sequencing projects that include subjects who do not have a definite diagnosis of TSC, need to be matched with correct variant interpretation. Two of our cases, neither of whom had a clinical diagnosis of TSC, had stop variants in exon 25 (*TSC2* c.2859dup, p.K954Qfs*6) and exon 31 (*TSC2* c.3837C>G, p.Y1279*), respectively, that would necessitate a diagnosis of TSC according to the updated TSC diagnostic criteria [Northrup et al., 2013]. This heightened our awareness to the downside of automatically associating stop codons in the alternatively spliced *TSC2* exons 25 and 31 with TSC.

Defining pathological as “causing TSC,” the only two exons in *TSC2* in which no pathogenic variants have been identified to date are exons 25 and 31 (www.lovd.nl/TSC2). Therefore, recent reports of “pathogenic” variants in these alternatively spliced exons led us to review different strands of evidence to try and ascertain the functional and clinical importance of these exons and of the putative “pathogenic” variants reported within them.

First, we examined amino acid sequences in exons 25 and 31 in multiple species to assess the conservation of these exons that may shed some light on their relevance to TSC. Although our simple and statistical analyses suggest that the conservation in these exons is less compared with the majority of the *TSC2* exons, the results still point to some importance for these exons, possibly in early development and/or specific tissues.

Next, we investigated the extent to which these exons undergo alternative splicing. As evident from available data on RNA expression in adult human tissues, there are tissue‐specific differences, but these support the abundance of the −ex25 and −ex31 transcripts (Supp. Figs. S4 and S5; Supp. Table S3). It was difficult to find data comparing the same human adult and fetal tissue types. In the mouse, the +ex25 transcript was more abundant in fetal and neonatal brain, whereas adult brain had an abundance of the −ex25 transcript [Xu et al., 1995; Olsson et al., 1996]. This agrees with tissue‐specific expression data showing predominantly the −ex25 transcript in adult human brain [https://goo.gl/6V7PpS] and could point to some relevance in stage‐specific development. More information on transcript and tissue‐specific expression of *TSC2* in the human fetus could help clarify the relative importance and specific functions of the different transcripts. Nonetheless, the evidence reviewed here leaves no doubt that *TSC2* transcripts lacking exon 25 or 31 exist and suggest that they form the majority of *TSC2* transcripts in a wide range of adult human tissues, including those, such as brain, important in TSC.

Finally, we investigated the influence of the amino acid sequences corresponding to exons 25 and 31 on TSC complex function. We used a previously described functional test that assays the effect of the TSC complex on TORC1 activity [Hoogeveen‐Westerveld et al., 2011, 2013]. According to this assay, the amino acids encoded by *TSC2* exons 25 and 31 are not required for TSC complex function. Furthermore, we did not obtain any evidence that the p.S1282_G1285delinsR or p.S1336L variants significantly affected TSC complex function. Also, none of the tested variants that map to exon 25 affect TSC complex function [Hoogeveen‐Westerveld et al., 2011, 2013].

Our analysis indicates that variants within *TSC2* exon 25 or 31 do not inactivate the TSC complex and are unlikely to cause TSC. Coincidentally, just prior to submission of this manuscript, a batch of data submitted to the TSC databases was found to contain the same exon 31 variant (*TSC2* c.3846_3855delinsG) as in case 3. This in‐frame deletion co‐occurs with a definite TSC‐causing variant in *TSC2* exon 5 (*TSC2* c.569dup, p.Y190*) in an infant who fulfils the diagnostic criteria for TSC (cortical tubers, SEGA, subependymal nodules, hypomelanotic macules, renal angiomyolipoma, epilepsy). The exon 5 and exon 31 variants (*TSC2* c.569dup and *TSC2* c.3846_3855delinsG) are both inherited from a parent who also has clinical TSC (hypomelanotic macules, facial angiofibromas, cortical tubers, epilepsy, intellectual disability, and renal angiomyolipomas). This new case is consistent with our assertion that *TSC2* c.3846_3855delinsG on its own is not the cause of classical TSC. Nonetheless, it is possible that variants mapping to *TSC2* exon 25 or 31 might have a significant role in disease. For example, a role for the *TSC2* c.2859dup (p.K954Qfs*6) and c.3846_3855delinsG (p.S1282_G1285delinsR) variants in epilepsy cannot be excluded. The *TSC2* c.3846_3855delinsG variant has been identified in several other individuals with infantile epilepsy who are without clinical features of TSC (Panzer, 2015, unpublished data). Exons 25 and 31 encode consensus sites of AKT and AMPK phosphorylation, respectively (http://scansite3.mit.edu) [Yaffe et al., 2001]. The TSC2 protein is inactivated by AKT phosphorylation [Manning et al., 2002] and activated by AMPK phosphorylation [Inoki et al., 2006]. It is possible that these exons alter the activity or response to stimuli of the TSC complex, particularly during fetal development. More sensitive assays of TSC complex activity are required to compare the activities of the different TSC2 protein splice isoforms and variants in more detail.

The findings reported here are specific to *TSC2* exons 25 and 31, as stop variants that definitely cause TSC have been identified in all other *TSC2* exons. Additional evidence that variants within exon 31 do not cause TSC comes from reports of three truncating variants (two frameshift and one nonsense) in the EVS and ExAC population databases (Supp. Table S4). Although phenotype information is not available, the population frequencies are quite high (1:532–1:1,443 individuals), which supports our conclusion that these truncating variants do not cause TSC. The exon 25 and 31 cases described here show the need to exercise caution when assigning pathogenicity, especially if the variant found has never been shown to cause TSC in a patient with a firm clinical diagnosis. This caution is probably reasonable in many other diseases.

## Contributors

R.E., S.P., and M.N. drafted the original manuscript. R.E. and S.P classified variants in the *TSC2* database. R.E., S.P., and W.E. analyzed available expression data. M.N., M.H‐W., and M.W. performed functional assessment. N.M., C.C., M.B., SergioP, and N.L.D. performed the conservation analysis. J.P., S.S., M.C.M., and R.N. provided patient information. V.P., M.F., S.E.H. provided whole‐exome sequencing information from the UK10K study. The final manuscript had contributions from all authors.

*Disclosure statement*: The authors have no conflicts of interest to declare.

## Patient consent

Written informed consent was obtained for genetic testing and the case reports comply with the regulations of the respective local research ethics committees.

## Web resources

Expression analysis of TSC2 transcripts:

NCBI *Homo sapiens* Annotation Release 107, https://goo.gl/6V7PpS (BodyMap2 and Fagerberg et al., 2014 data showing the tracks for brain tissue; accessed November 2015).

NCBI *Homo sapiens* Annotation Release 107, https://goo.gl/Gu0JWq (Fagerberg et al., 2014 data; accessed November 2015).

## Supporting information

Disclaimer: Supplementary materials have been peer‐reviewed but not copyedited.

Supporting information

## References

[humu22951-bib-0001] Adzhubei I , Jordan DM , Sunyaev SR . 2013 Predicting functional effect of human missense mutations using PolyPhen‐2. Curr Protoc Hum Genet. Chapter 7:Unit7.20. 10.1002/0471142905.hg0720s76 PMC4480630 23315928

[humu22951-bib-0002] Au KS , Williams AT , Roach ES , Batchelor L , Sparagana SP , Delgado MR , Wheless JW , Baumgartner JE , Roa BB , Wilson CM , Smith‐Knuppel TK , Cheung MY , et al. 2007 Genotype/phenotype correlation in 325 individuals referred for a diagnosis of tuberous sclerosis complex in the United States. Genet Med 9:88–100. 17304050 10.1097/gim.0b013e31803068c7

[humu22951-bib-0003] Dibble CC , Manning BD . 2013 Signal integration by mTORC1 coordinates nutrient input with biosynthetic output. Nat Cell Biol 15:555–564. 23728461 10.1038/ncb2763 PMC3743096

[humu22951-bib-0004] Dunlop EA , Dodd KM , Land SC , Davies PA , Martins N , Stuart H , McKee S , Kingswood C , Saggar A , Corderio I , Duarte Medeira AM , Kingston H et al. 2011 Determining the pathogenicity of patient‐derived TSC2 mutations by functional characterization and clinical evidence. Eur J Hum Genet 19:789–795. 21407264 10.1038/ejhg.2011.38 PMC3137505

[humu22951-bib-0005] Eggington JM , Bowles KR , Moyes K , Manley S , Esterling L , Sizemore S , Rosenthal E , Theisen A , Saam J , Arnell C , Pruss D , Bennett J , et al. 2014 A comprehensive laboratory‐based program for classification of variants of uncertain significance in hereditary cancer genes. Clin Genet 86:229–237. 24304220 10.1111/cge.12315

[humu22951-bib-0006] European Chromosome 16 Tuberous Sclerosis Consortium . 1993 Identification and characterization of the tuberous sclerosis gene on chromosome 16. Cell 75:1305–1315. 8269512 10.1016/0092-8674(93)90618-z

[humu22951-bib-0007] Fagerberg L , Hallström BM , Oksvold P , Kampf C , Djureinovic D , Odeberg J , Habuka M , Tahmasebpoor S , Danielsson A , Edlund K , Asplund A , Sjöstedt E , et al. 2014 Analysis of the human tissue‐specific expression by genome‐wide integration of transcriptomics and antibody‐based proteomics. Mol Cell Proteomics 13:397–406. 24309898 10.1074/mcp.M113.035600 PMC3916642

[humu22951-bib-0008] GTEx Consortium . 2015 Human genomics. The genotype‐tissue expression (GTEx) pilot analysis: multitissue gene regulation in humans. Science 348:648–660. 25954001 10.1126/science.1262110 PMC4547484

[humu22951-bib-0009] Hoogeveen‐Westerveld M , Wentink M , van den Heuvel D , Mozaffari M , Ekong R , Povey S , den Dunnen JT , Metcalfe K , Vallee S , Krueger S , Bergoffen J , Shashi V , et al. 2011 Functional assessment of variants in the TSC1 and TSC2 genes identified in individuals with tuberous sclerosis complex. Hum Mutat 32:424–435. 21309039 10.1002/humu.21451

[humu22951-bib-0010] Hoogeveen‐Westerveld M , Ekong R , Povey S , Mayer K , Lannoy N , Elmslie F , Bebin M , Dies K , Thompson C , Sparagana SP , Davies P , van den Ouweland A , et al. 2013 Functional assessment of TSC2 variants identified in individuals with tuberous sclerosis complex. Hum Mutat 34:167–175. 22903760 10.1002/humu.22202

[humu22951-bib-0011] Inoki K , Ouyang H , Zhu T , Lindvall C , Wang Y , Zhang X , Yang Q , Bennett C , Harada Y , Stankunas K , Wang CY , He X , et al. 2006 TSC2 integrates Wnt and energy signals via a coordinated phosphorylation by AMPK and GSK3 to regulate cell growth. Cell 126:955–968. 16959574 10.1016/j.cell.2006.06.055

[humu22951-bib-0012] Jansen AC , Sancak O , D'Agostino MD , Badhwar A , Roberts P , Gobbi G , Wilkinson R , Melanson D , Tampieri D , Koenekoop R , Gans M , Maat‐Kievit A , et al. 2006 Unusually mild tuberous sclerosis phenotype is associated with TSC2 R905Q mutation. Ann Neurol 60:528–539. 17120248 10.1002/ana.21037

[humu22951-bib-0013] Khare L , Strizheva GD , Bailey JN , Au KS , Northrup H , Smith M , Smalley SL , Henske EP . 2001 A novel missense mutation in the GTPase activating protein homology region of TSC2 in two large families with tuberous sclerosis complex. J Med Genet 38:347–349. 11403047 10.1136/jmg.38.5.347 PMC1734876

[humu22951-bib-0014] Kimura K , Wakamatsu A , Suzuki Y , Ota T , Nishikawa T , Yamashita R , Yamamoto J , Sekine M , Tsuritani K , Wakaguri H , Ishii S , Sugiyama T , et al. 2006 Diversification of transcriptional modulation: large‐scale identification and characterization of putative alternative promoters of human genes. Genome Res 16:55–65. 16344560 10.1101/gr.4039406 PMC1356129

[humu22951-bib-0015] Manning BD , Tee AR , Logsdon MN , Blenis J , Cantley LC . 2002 Identification of the tuberous sclerosis complex‐2 tumor suppressor gene product tuberin as a target of the phosphoinositide 3‐kinase/akt pathway. Mol Cell 10:151–162. 12150915 10.1016/s1097-2765(02)00568-3

[humu22951-bib-0016] Mayer K , Goedbloed M , van Zijl K , Nellist M , Rott HD . 2004 Characterisation of a novel TSC2 missense mutation in the GAP related domain associated with minimal clinical manifestations of tuberous sclerosis. J Med Genet. 4:e64. 15121792 10.1136/jmg.2003.010835 PMC1735780

[humu22951-bib-0017] Nellist M , Sancak O , Goedbloed MA , Rohe C , van Netten D , Mayer K , Tucker‐Williams A , van den Ouweland AM , Halley DJ . 2005 Distinct effects of single amino‐acid changes to tuberin on the function of the tuberin‐hamartin complex. Eur J Hum Genet 13:59–68. 15483652 10.1038/sj.ejhg.5201276

[humu22951-bib-0018] Ng PC , Henikoff S . 2003 SIFT: Predicting amino acid changes that affect protein function. Nucleic Acids Res 31:3812–3814. 12824425 10.1093/nar/gkg509 PMC168916

[humu22951-bib-0019] Northrup H , Krueger DA ; International Tuberous Sclerosis Complex Consensus Group . 2013 Tuberous sclerosis complex diagnostic criteria update: recommendations of the 2012 International Tuberous Sclerosis Complex Consensus Conference. Pediatr Neurol 49:243–254. 24053982 10.1016/j.pediatrneurol.2013.08.001 PMC4080684

[humu22951-bib-0020] O'Connor SE , Kwiatkowski DJ , Roberts PS , Wollmann RL , Huttenlocher PR . 2003 A family with seizures and minor features of tuberous sclerosis and a novel TSC2 mutation. Neurology 61:409–412. 12913212 10.1212/01.wnl.0000073272.47681.bb

[humu22951-bib-0021] Olsson PG , Schofield JN , Edwards YH , Frischauf AM . 1996 Expression and differential splicing of the mouse TSC2 homolog. Mamm Genome 7:212–215. 8833243 10.1007/s003359900057

[humu22951-bib-0022] Osborne JP , Fryer A , Webb D . 1991 Epidemiology of tuberous sclerosis. Ann NY Acad Sci. 615:125–127. 2039137 10.1111/j.1749-6632.1991.tb37754.x

[humu22951-bib-0023] R Core Team . 2015 R: A language and environment for statistical computing. R Foundation for Statistical Computing Vienna, Austria Accessed at: https://www.R‐project.org/.

[humu22951-bib-0024] Sampson JR , Scahill SJ , Stephenson JB , Mann L , Connor JM . 1989 Genetic aspects of tuberous sclerosis in the west of Scotland. J Med Genet 26:28–31. 2918523 10.1136/jmg.26.1.28 PMC1015532

[humu22951-bib-0025] Sancak O , Nellist M , Goedbloed M , Elfferich P , Wouters C , Maat‐Kievit A , Zonnenberg B , Verhoef S , Halley D , van den Ouweland A . 2005 Mutational analysis of the TSC1 and TSC2 genes in a diagnostic setting: genotype–phenotype correlations and comparison of diagnostic DNA techniques in tuberous sclerosis complex. Eur J Hum Genet 13:731–741. 15798777 10.1038/sj.ejhg.5201402

[humu22951-bib-0026] Schaaf CP , Sabo A , Sakai Y , Crosby J , Muzny D , Hawes A , Lewis L , Akbar H , Varghese R , Boerwinkle E , Gibbs RA , Zoghbi HY . 2011 Oligogenic heterozygosity in individuals with high‐functioning autism spectrum disorders. Hum Mol Genet 20:3366–3375. 21624971 10.1093/hmg/ddr243 PMC3153303

[humu22951-bib-0027] UK10K Consortium , Walter K , Min JL , Huang J , Crooks L , Memari Y , McCarthy S , Perry JR , Xu C , Futema M , Lawson D , Iotchkova V , et al. 2015 The UK10K project identifies rare variants in health and disease. Nature 526:82–90. 26367797 10.1038/nature14962 PMC4773891

[humu22951-bib-0028] Valdar WS. 2002 Scoring residue conservation. Proteins 48:227–241. 12112692 10.1002/prot.10146

[humu22951-bib-0029] Wentink M , Nellist M , Hoogeveen‐Westerveld M , Zonnenberg B , van der Kolk D , van Essen T , Park S‐M , Woods G , Cohn‐Hokke P , Brussel W , Smeets E , Brooks A , et al. 2012 Functional characterization of the TSC2 c.3598C>T (p.R1200W) missense mutation that co‐segregates with tuberous sclerosis complex in mildly affected kindreds. Clin Genet 81:453–461. 21332470 10.1111/j.1399-0004.2011.01648.x

[humu22951-bib-0030] Xiao GH , Jin F , Yeung RS . 1995 Identification of tuberous sclerosis 2 messenger RNA splice variants that are conserved and differentially expressed in rat and human tissues. Cell Growth Differ 6:1185–1191. 8519695

[humu22951-bib-0031] Xu L , Sterner C , Maheshwar MM , Wilson PJ , Nellist M , Short PM , Haines JL , Sampson JR , Ramesh V . 1995 Alternative splicing of the tuberous sclerosis 2 (TSC2) gene in human and mouse tissues. Genomics 27:475–480. 7558029 10.1006/geno.1995.1079

[humu22951-bib-0032] Yaffe MB , Leparc GG , Lai J , Obata T , Volinia S , Cantley LC . 2001 A motif‐based profile scanning approach for genome‐wide prediction of signaling pathways. Nat Biotechnol 19:348–353. 11283593 10.1038/86737

