# Supplementary material for: Variants Within TSC2 Exons 25 and 31 Are Very Unlikely to Cause Clinically Diagnosable Tuberous Sclerosis
Source: Hum Mutat. 2016 Jan 12;37(4):364–70. doi: 10.1002/humu.22951 (PMC4843954; doi:10.1002/humu.22951)
Supplement: Supplementary file 1 — Supporting information [file HUMU-37-364-s001.pdf]

SUPPORTING INFORMATION

Supp. Figure S1. Alignment of amino acid sequences encoded by exons 25 and 31 in *TSC2* orthologs. Manual alignment of protein sequences corresponding to TSC2 exons 25 and 31 from 163 vertebrate species, showing conservation down to the lamprey. **(A)** Exon 25 alignment. **(B)** Exon 31 alignment. *A few orthologues lack sequences for the exons; two in exon 25 and four in exon 31 (light-brown highlighted dots). **The two-letter codes of the 163 vertebrates in the alignment. Amino acid variants highlighted in yellow on the first line and in the alignment have been reported to the TSC2 LOVD; amino acid positions in the human sequence are also indicated and there are different lines of evidence to support the "no pathogenicity" classification. Variants without colours at positions 954, 960 and 984 are of "uncertain clinical significance". The most conserved amino acids in vertebrates are highlighted in magenta; those highlighted in blue are the most conserved amino acids in fishes. Four amino acids (HHLH) in exon 25 of Esox lucius (Ex), represented by // in the alignment, were removed due to the lack of space.

The amino acid numbering in the alignment corresponds to the human *TSC2* cDNA sequence (GenBank NM_000548.3, GI:116256351) with the translation initiation codon as +1.

Supp. Figure S1 legend (continued)

🟡 = Complete *TSC2* Sequence (>98%) 🟢 = Partial *TSC2* Sequence

Primates	**Hominoidea (apes)**	Hs	Homo sapiens	🟡
		Pa	Pan paniscus (pygmy chimpanzee)	🟡
		Pt	Pan troglodytes (chimpanzee)	🟡
		Go	Gorilla gorilla	🟡
		Ou	Pongo abelii (orangutan)	🟡
		Nl	Nomascus leucogenys (gibbon)	🟡
	**Old World monkey (Cercopithecidae)**	Rh	Rhinopithecus roxellana (golden snub-nosed monkey)	🟡
		Cq	Colobus angolensis (black-and-white colobus monkey)	🟡
		Nm	Nasalis larvatus (proboscis monkey)	🟢
		Rm	Macaca mulatta (Rhesus monkey)	🟡
		Ph	Papio hamadryas (baboon)	🟡
		Hu	Chlorocebus sabaeus (green monkey)	🟡
	**New World monkeys (Simiiformes)**	Sb	Saimiri boliviensis (squirrel monkey)	🟡
		Au	Aotus nancymaae (Ma's night monkey)	🟡
		Cj	Callithrix jacchus (white-tufted-ear marmoset)	🟡
	**Strepsirrhini**	Og	Otolemur garnettii (small-eared galago, lemur)	🟡
		Le	Microcebus murinus (gray mouse lemur)	🟡
		Pq	Propithecus coquereli (Coquerel's sifaka, lemur)	🟡
		Ua	Daubentonia madagascariensis (aye-aye lemur)	🟡
	**Platyrrhini**	Tr	Tarsius syrichta (Philippine tarsier)	🟡
	**Dermoptera**	Gv	Galeopterus variegatus (Sunda flying lemur)	🟡
	**Laurasiatheria**	Ec	Equus caballus (horse)	🟡
		Rc	Ceratotherium simum simum (southern white rhinoceros)	🟡
		La	Vicugna pacos (alpaca)	🟡
		Lu	Camelus ferus (wild Bactrian camel)	🟡
		Cb	Camelus bactrianus (Bactrian camel)	🟡
		Ld	Camelus dromedarius (Arabian camel)	🟡
		Cf	Canis lupus familiaris (dog)	🟡
		Tg	Panthera tigris altaica (Amur tiger)	🟡
		Fc	Felis catus (domestic cat)	🟡
		Fu	Mustela putorius furo (domestic ferret)	🟡
		Us	Ursus maritimus (polar bear)	🟡
		Pe	Ailuropoda melanoleuca (giant panda)	🟡
		Fa	Leptonychotes weddellii (Antarctic Weddell seal)	🟡
		Ob	Odobenus rosmarus rosmarus (Atlantic walrus. Trichechus)	🟡
		Bt	Bos taurus (cattle)	🟡
		Ce	Capreolus capreolus (Europian roe deer)	🟡
		Ka	Capra hircus (inner Mongolia Cashmere)	🟡
		At	Pantholops hodgsonii (Tibetan antelope. Chiru)	🟢
		Oa	Ovis aries (sheep)	🟡
		Ss	Sus scrofa (pig)	🟡
		Tt	Tursiops truncatus (bottlenosed dolphin)	🟡
		Lx	Lipotes vexillifer (Yangtze river dolphin)	🟡
		Ro	Orcinus orca (killer whale)	🟡
		Ba	Balaenoptera acutorostrata scammoni (minke whale)	🟡
		Co	Physeter catodon (sperm whale)	🟡
		My	Myotis lucifugus (little brown bat)	🟡
		Tf	Eptesicus fuscus (big brown bat)	🟡
		Pv	Pteropus vampyrus (large flying fox, bat)	🟡
		Mp	Manis pentadactyla (Chinese pangolin)	🟡
	**Lagomorpha**	Oc	Oryctolagus cuniculus (rabbit)	🟡
		Pk	Ochotona princeps (American pika)	🟡
	**Scandentia**	Tp	Tupaia chinensis (Chinese tree shrew)	🟡
	**Rodentia**	In	Chinchilla lanigera (long-tailed chinchilla)	🟡
		St	Ictidomys tridecemlineatus (thirteen-lined ground squirrel)	🟡
		Mk	Marmota marmota (Alpine marmot)	🟡
		Oe	Octodon degus (degu, brush-tailed rat)	🟡
		Cv	Cavia porcellus (domestic guinea pig)	🟡
		Hg	Heterocephalus glaber (naked mole-rat)	🟡
		Fd	Fukomys damarensis (Damara mole-rat)	🟡
		Pm	Peromyscus maniculatus (rodent, deer mouse)	🟡
		Cg	Cricetulus griseus (Chinese hamster)	🟡
		Mm	Mus musculus (house mouse)	🟡

	Mc	Microtus ochrogaster (prairie vole)
	Rn	Rattus norvegicus
	Jj	Jaculus jaculus (lesser Egyptian jerboa)
	Ng	Nannospalax galili (upper Galilee mountains, blind mole rat)
	Do	Dipodomys ordii (Ord's kangaroo rat)
**Laurasiatheria**	Sa	Sorex araneus (European shrew)
	Ee	Erinaceus europaeus (western European hedgehog)
**Afrotheria**	Et	Echinops telfairi (small Madagascar hedgehog)
	Ew	Elephantulus edwardii (Cape rock elephant shrew)
	Ry	Chrysochloris asiatica (Cape golden mole)
	Pr	Procavia capensis (Cape rock hyrax)
	El	Loxodonta africana (African savanna elephant)
	Mn	Trichechus manatus latirostris (Florida manatee)
	Of	Orycteropus afer afer (giant anteater)
**Xenarthra**	Ch	Choloepus hoffmanni (bradipus, Hoffmann's two-fingered sloth)
	Ar	Dasypus novemcinctus (nine-banded armadillo)
**Metatheria, Marsupials**	Sh	Sarcophilus harrisii (marsupial, tasmanian devil)
	Cu	Macropus eugenii (marsupial, tammar wallaby)
	Op	Monodelphis domestica (marsupial, gray short-tailed opossum)
**Monotremata, Platypus**	On	Ornithorhynchus anatinus (platypus, monotremata)
**Sauria, Birds**	Gg	Gallus gallus (chicken)
	Me	Meleagris gallopavo (turkey)
	Ai	Anas platyrhynchos (mallard duck)
	Cl	Columba livia (rock pigeon)
	Ep	Aptenodytes forsteri (emperor penguin)
	Ae	Struthio camelus (African ostrich)
	Mu	Melopsittacus undulatus (budgerigar)
	Ps	Taeniopygia guttata (zebra finch)
	Uh	Pseudopodoces humilis (Tibetan ground-tit)
**Sauria, Reptiles, Crocodils**	Cm	Alligator mississipiensis
	As	Alligator sinensis
	Gn	Gavialis gangeticus (alligator)
	Ck	Crocodylus porosus (Australian saltwater crocodile)
**Sauria, Reptiles, Turtles**	Cd	Chelonia mydas (green seaturtle)
	Te	Chrysemys picta bellii (painted turtle)
	If	Apalone spinifera (spiny softshell turtle)
	Tl	Pelodiscus sinensis (softshell turtle)
**Sauria, Reptiles, Lizard**	Ac	Anolis carolinensis (lizard, iguana)
**Sauria, Reptiles, Snakes**	Pd	Python molurus (Indian rock python)
	Tk	Thamnophis sirtalis (common garter snake)
	Oh	Ophiophagus hannah (king cobra)
	Cr	Crotalus mitchellii pyrrhus (speckled rattlesnake)
	Vb	Vipera berus berus (common viper)
**Amphibia, Frogs**	Ug	Pseudacris regilla (Pacific treefrog)
	Rx	Rana clamitans (bronze frog)
	Np	Nanorana parkeri (high Himalaya frog)
	Xt	Xenopus tropicalis (clawed frog)
**Amphibia, Salamanders**	Hc	Hynobius chinensis (Chinese salamander)
	Sx	Ambystoma mexicanum (axolotl)
	Cy	Cynops pyrrhogaster (Japanese firebelly newt)
	Sv	Notophthalmus viridescens (eastern newt)
**Cartilaginous fishes**	Rz	Leucoraja erinacea (little skate)
	Ef	Callorhinchus milii (elephant shark)
**Bony fishes**	Li	Latimeria chalumnae (coelacanth)
	Lo	Lepisosteus oculatus (spotted garfish)
	Eh	Apteronotus leptorhynchus (brown ghost knifefish)
	Ax	Astyanax mexicanus (Mexican tetra / blind cavefish)
	Gf	Scleropages formosus (Asian bonytongue)
	Aj	Anguilla japonica (Japanese eel)
	Qh	Clupea harengus (Atlantic herring)
	Gi	Ctenopharyngodon idella (grass carp)
	Pp	Pimephales promelas (fathead minnow)
	Dr	Danio rerio (zebrafish)
	Ta	Oncorhynchus mykiss (rainbow trout)
	Se	Salmo salar (Atlantic salmon)
	Ex	Esox lucius (northern pike)
	Ol	Oryzias latipes (Japanese medaka)
	Gd	Gadus morhua (Atlantic cod)
	Pw	Periophthalmodon schlosseri (giant mudskipper)
	Pg	Periophthalmus magnuspinnatus (mudskipper)

	Bp	Boleophthalmus pectinirostris (great blue-spotted mudskipper)
	Sq	Scartelaos histophorus (walking goby)
	Hi	Channa striata (snakehead murrel)
	Sl	Cynoglossus semilaevis (tangue sole)
	Vi	Solea senegalensis
	Nf	Anoplopoma fimbria (sablefish)
	Lr	Larimichthys crocea (large yellow croaker)
	Ni	Notothenia coriiceps (black rockcod/Arctic fish)
	Sw	Stegastes partitus (bicolor damselfish)
	Dl	Dicentrarchus labrax (European seabass)
	Er	Sebastes rubrivinctus (flag rockfish)
	Qa	Pampus argenteus (silver pomfret)
	Ab	Amphilophus citrinellus (Midas cichlid)
	Ht	Thunnus orientalis (Pacific bluefin tuna)
	Ow	Pseudopleuronectes yokohamae (marbled flounder)
	Ff	Poecilia formosa (Amazon molly)
	Xm	Xiphophorus maculatus (southern platyfish)
	Cz	Cyprinodon variegatus (Sheepshead minnow)
	Ga	Gasterosteus aculeatus (three-spined stickleback)
	Ot	Oreochromis niloticus (Nile tilapia)
	Hx	Haplochromis burtoni (Burton's mouthbrooder)
	Pj	Pundamilia nyererei (cichlid endemic to Lake Victoria)
	Nb	Neolamprologus brichardi (lyretail cichlid)
	Mz	Maylandia zebra (zebra mbuna)
	Fr	Takifugu rubripes (torafugu)
	Nh	Nothobranchius furzeri (turquoise killifish)
	Ul	Austrofundulus limnaeus (inhabit ephemeral ponds, killifish)
	Tn	Tetraodon nigroviridis (spotted green pufferfish)
**Cyclostomata, Lampreys**	Lc	Lethenteron camtschaticum (Arctic lamprey)
	Lp	Petromyzon marinus (lamprey)

Supp. Figure S1A-B

(A) 161 alignments * — /----------------------exon 25---------------------/

(B) 159 alignments* — /-----------exon 31-----------/

```
                                      963
                                    961
         954                       960                           988
      951                        958             972         984  P        1276                1282      1291
       S  R                        S FL M          T           Q  C   **       F                   G        I
                                                                                                   ---
SLRIARPPKQ-------------GLNNSPPVKEFKESSAAEAFRCRSISVSEHVVR  Hs  VASFSS---------LYQSSCQGQLHRSVSWA
SLRIARPPKQ-------------GLNNSPPVKEFKESSAAEAFRCRSISVSEHVVR  Pa  VASFSS---------LYQSSCQGQLHRSVSWA
SLRIARPPKQ-------------GLNNSPPVKEFKESSAAEAFRCRSISVSEHVVR  Pt  VASFSS---------LYQSSCQGQLHRSVSWA
SLRIARPPKQ-------------GLNNSPPVKEFKESSAAEAFRCRSISVSEHVVR  Go  VASFSS---------LYQSSCQGQLHRSVSWA
SLRIARPPKQ-------------GLNNSPPVKEFKESSAAEAFRCRSISVSEHVVR  Ou  VASFSS---------LYQSSCQGQLHRSVSWA
SLRIARPPKQ-------------GLNNSPPVKEFKESSAAEAFRCRSISVSEHVVR  Nl  VASFSS---------LYQSSCQGQLHRSVSWA
SLRIARPPKQ-------------GLNNSPPVKEFKESSAAEAFRCRSISVSEHVVR  Rh  VASFSS---------LYQSSCQGQLHRSVSWA
SLRIARPPKQ-------------GLNNSPPVKEFKESSAAEAFRCRSISVSEHVVR  Cq  VASFSS---------LYQSSCQGQLHRSVSWA
SLRIARPPKQ-------------GLNNSPPVKEFKESSAAEAFRCRSISVSEHVVR  Nm  VASFSS---------LYQSSCQGQLHRSVSWA
SLRIARPPKQ-------------GLNNSPPVKEFKESSAAEAFRCRSISVSEHVVR  Rm  VASFSS---------LYQSSCQGQLYRSVSWA
SLRIARPPKQ-------------GLNNSPPVKEFKESSAAEAFRCRSISVSEHVVR  Ph  VASFSS---------LYQSSCQGQLHRSVSWA
SLRIARPPKQ-------------GLNNSPPVKEFKESSAAEAFRCRSISVSEHVVR  Hu  VASFSS---------LYQSSCQGQLHRSVSWA
SLRIARPPKQ-------------GLNNSPPVKEFKESSAAEAFRCRSISVSEHVVR  Sb  VASFSS---------LYQSGCQGQLHRSVSWA
SLRIARPPKQ-------------GLNNSPPVKEFKESSAAEAFRCRSISVSEHVVR  Au  VASFSS---------LYQSSCQGQLHRSISWA
SLRIARPPKQ-------------GLNNSPPVKEFKESSAAEAFRCRSISVSEHVVR  Cj  VASFSS---------LYQSSCQGQLHRSISWA
SLRIARPPKQ-------------GLNNSPPVKESKESCAAEAFRCRSISVSDHVVH  Og  VASFSS---------LYQSSCQGKLHRSISWA
SLRIARPPKQ-------------GLNNSPPVKESKESCASEAFRCRSISVSDHAVH  Le  VASFSS---------LYQSSCQGKLHRSISWA
SLRIARPPKQ-------------GLNNSPPVKESKESCASEAFRCRSISVSDHAVH  Pq  VASFSS---------LYQSSCQGKLHRSISWA
SLRIARPPKQ-------------GLNNSPPVKESKESCASEAFRCRSISVSDHVVH  Ua  VASFSS---------LYQSSCQGKLHRSISWA
SLRIARPPKQ-------------GLNNSPPVKEFKESSAAEAFRCRSISVSEHVVR  Tr  VASFSS---------LCQSSCQGKLHRSISWA
SLRIARPPKQ-------------GWNNSPPVKECKESSAAEAFRCRSISVSEHVVR  Gv  VASFSS---------LYQSSCQGKLHRSISWA
SLRIARPPKQ-------------GLNNSPPVKEFKESSAADAFRCRSISVSEHVVR  Ec  VASFFS---------LYQSSCQGKLHRSISWA
SLRIARPPKQ-------------GLNNSPPVKEFKESSAADAFRCRSISVSEHVVR  Rc  VASFSS---------LYQSSCQGKLHRSISWA
SLRIARPPKQ-------------GLNNSPPVKEFKESSAADAFRCRSISVSEHVVR  La  VASFSS---------LYQSSCQRKLHRSISWA
SLRIARPPKQ-------------GLNNSPPVKEFKESSAADAFRCRSISVSEHVVR  Lu  VASFSS---------LYQSSCQGKLHRSISWA
SLRIARPPKQ-------------GLNNSPPVKEFKESSAADAFRCRSISVSEHVVR  Cb  VASFSS---------LYQSSCQGKLHRSISWA
SLRIARPPKQ-------------GLNNSPPVKEFKESSAADAFRCRSISVSEHVVR  Ld  VASFSS---------LYQSSCQGKLHRSISWA
SLRIARPPKQ-------------GLNNSPPVKEFKESSAAEAFRCRSISVSEHVVR  Cf  VASFSS---------LYQSSCQGKLHRSISWA
SLRIARPPKQ-------------GLNNSPPVREFQESSAADAFRCRSISVSEHVVR  Tg  VASFSS---------LYQTSCQGELHRSISWA
SLRIARPPKQ-------------GLNNSPPVREFQESSAADAFRCRSISVSEHVVR  Fc  VASFSS---------LYQTSCQGELHRSISWA
SLRIARTPKQ-------------GLTNSPPVKECKESSAAEAFRCRSISVSEHVVR  Fu  VASLSS---------LCQSSCRAQLHRGVSWA
SLRIARPPKQ-------------GLNNSPPVKEFKESSAAEAFRCRSISVSEHAVR  Us  VASFSS---------LCQSSCQAKLHRTISWA
SLRIARPPKQ-------------GLNNSPPVKEFKESSAAEAFRCRSVSVSEHAVR  Pe  VASFSS---------LCQSSCQAKLHRTISWA
SLRIARPPKQ-------------GLNNSPPVKEFKESPAAEAFRCRSISVSEHVVR  Fa  VASFSS---------LYQSSCQAKLHRSISWA
SLRIARPPKQ-------------GLNNSPPVKEFKESPAAEAFRCRSISVSEHVVR  Ob  VASFSS---------LCQSSCQAKLHRSISWA
SLRIARPPKQ-------------GLNNSPPVKELKESSAADAFRCRSISVSEHVVR  Bt  VASFSS---------LCQSRCQGKLHRSISWA
SLRIARPPKQ-------------GLNNSPPVKELKESSAADAFRCRSISVSEHVVR  Ce  VASFSS---------LCQSRCQGRLHRSISWA
SLRIARPPKQ-------------GLNNSPPVKELQESSAADAFRCRSVSVSERVVR  Ka  VASFSS---------LCQSRCQGKLHRSISWA
SLRIARPPKQ-------------GLNNSPPVKELKESSAADAFRCRSISVSEHVVR  At  VASFSS---------LCQSRCQGKLHRSISWA
SLRIARPPKQ-------------GLNNSPPVKELKESSAADAFRCRSISVSEHVVR  Oa  VASFSS---------LCQSRCQGKLHRSISWA
SLRIARPPKQ-------------GLNNSPPVKEFKESLAADAFRCRSISVSEHAVR  Ss  VASFSS---------LCQSRCQGKLHRSISWA
SLRIARPPKQ-------------GLNNSPPVKEFKESSAADAFRCRSISVSEHVVR  Tt  VASFSS---------LYQSSCQGKLHRSISWA
SLRIARPPKQ-------------GLNNSPPVKEFKESSAADAFRCRSISVSEHVVR  Lx  VASFSS---------LYQSSCQGKLHRSISWA
SLRIARPPKQ-------------GLNNSPPVKEFKESSAADAFRCRSISVSEHVVR  Ro  VASFSS---------LYQSSCQGKLHRSISWA
SLRIARPPKQ-------------GLNNSPPVKEFKESSAADAFRCRSISVSEHVVR  Ba  VASFSS---------LYQSSCQGKLHRSISWA
SLRIARPPKQ-------------GLNNSPPVKEFKESSAADAFRCRSISVSEHVVR  Co  VASFSS---------LYQSSCQGKLHRSISWA
SLRIARPPKQ-------------GLNNSPPVKEFKESSAADAFRCRSISVSEHVVR  My  VASFSP---------LYQSSCQGRLHRSISWA
SLRIARPPKQ-------------GLNNSPPVKEFKESSAADAFRCRSISVSEHVVR  Tf  VASFLS---------LYQSSCQGKLRRSISWA
SLRIARPPKQ-------------GLNNSPPVKEFKESSAADAFRCRSISVSEHVVR  Pv  VASFSS---------LYQSSCQGKLHRSISWA
SLRIARAPKQ-------------GLNGSPPVKEFKESSAADAFRCRSISVSEHVVR  Mp  VASFSS---------LYQSSCHGKLHRSISWA
SLRIARAPRP-------------GLAHSPPVDECKGSPAAEAFRCRSVSVSEHGVR  Oc  VASLSS---------LCPSSCQGKLHRSISWA
SLRIARPPRQ-------------GLTHSPPMEECEGSPTAEAFRCRSVSVSEHAVR  Pk  VASLSS---------LCPSGCQGKLHRSISWA
SVRIARPPKH-------------GSSHSPPVREREESAASEAFRCRSVSVSERVVR  Tp  VASLSS---------LCPAGCPGALRRSVSWA
SLRIARAPKQ-------------GLNNSPPVKEFKESSAAEAFRCRSISVSEHVVR  In  VASFSS---------LYQSSCQGQLHRSISWA
SLRIARAPKQ-------------GSNNSPPVKELKESPAAEAFRCRSVSVSDHVVR  St  VASFSS---------LYQSSCQGQLHRSISWA
SLRIARAPKQ-------------GSNNSPPVKELKESPAAEAFRCRSVSVSDHVVR  Mk  VASFSS---------LYQSSCQGQLHRSISWA
SLRIARAPKQ-------------GLNNSPPVKEFKESSAAEAFRCRSISVSEHVVR  Oe  VASFSS---------LYQSSCQGQLHRSISWA
SLRIARAPKQ-------------GLNNSPPVKEFKESSAAEAFRCRSISVSEHVVR  Cv  VASFSS---------LYQSSCQGQLHRSISWA
```

```
SLRIARAPKQ-------------GLNNSPPVKEFKESSAAEAFRCRSISVSEHVVR  Hg  VASFSS---------LYQSNCQGQLHRSISWA
SLRIARAPKQ-------------GLNNSPPVKEFKESSAAEAFRCRSISVSEHVVR  Fd  VASFSS---------LYQSTCQGQLHRSISWA
SLRIARAPKQ-------------GLNNSPPVKEFRESCAAETFRCRSISVSEHVVR  Pm  VASFSS---------LYQPSCQGQLHRSVSWA
SLRIARVPKQ-------------GLNNSPPVKEFKESCAAEAFRCRSISVSEHVVR  Cg  VASFSS---------LYQPSCQGQLHRSVSWA
SLRIARAPKQ-------------GLNNSPPVKEFKESCAAEAFRCRSISVSEHVVR  Mm  VASFSS---------LYQPSCQGQLHRSVSWA
SLRIARAPKQ-------------GLNNSAPVKELRESCAAEAFRCRSVSVSEHVVR  Mc  VASFSS---------LYQSSCQGQLHRSVSWA
SLRIARAPKQ-------------GLNNSPPVKEFKESCAAEAFRCRSISVSEHVVR  Rn  VASFSS---------LYQPSCQGQLHRSVSWA
SLRIARAPRQ-------------GLNNSPPVKELKESCAAEAFRCRSISVSEHVVR  Jj  VASFSS---------LYQSSCQGQLHRSVSWA
SLRIARAPKQ-------------GLNNSPPVKEFKESCAAEAFRCRSISVSEHVVR  Ng  ................................
SLRIARAPKQ-------------GLNNSPPVKEFKESCAAEAFRCRSISVSEHVVR  Do  VASLSS---------LYQSSCQGQLHRSVSWA
SLRVARPPRQ-------------GLSHSPPVKELPESCAAEAFRCRSISVSEQVVR  Sa  ................................
SLRVARPPKQ-------------GLNNSPPVKEFKESSAAEVFRCRSISVSEHVVR  Ee  VASLSS---------LCQSSCRGRLHRSVSWA
SVRPARSPKQ-------------GSSHSRPVRERPESSAAEAFRCRSVSVSEQVVR  Et  VASLSS---------LSQSSCQGRLHRSVSWA
SLRLARPPRQ-------------GSSHSPPVNE-RKSCAAEAFRCRSISVSEQVVR  Ew  VASLSS---------LSPSSCRGKLHRSISWA
SLRPARPPKQ-------------GPTHSPPVKERKEGCAAEVFRCRSISVSEQAVR  Ry  VASFSS---------LSPSSGQGKLHRSVSWA
SVRVAGPPRQ-------------GLSNSPPVKERKGSGAAEAFRCRSISVSEQVVR  Pr  VASFSS---------LSQSSCQGKLHRSVSWA
SLKIARPPKQ-------------DLSNSPLVKECKESCASEAFQCHSISVSEQVVC  El  VASFSS---------LSQSSCQGKLHRSVSWA
SLRIARPPRQ-------------GSSNSLPVKKHEESCAAEAFRGRSISVSEQVVR  Mn  VASFSS---------LSQSSCQGKLHRSVSWA
SLRIARPLRQ-------------GPSNSAPGKERQESCAAEAFRCRSISVSEQVVR  Of  VASFCS---------LSQSSCRGKLHRSVSWA
SVRIARAPRQ-------------GLATSPPVKEVQESSA-EAFRCRSVSVSEHAAR  Ch  VASLSS---------LYQPSCQGELHRSISWA
SVRIARPPRQ-------------GLNNSPPAKELKESSAAEAFRCRSVSVSDHVAR  Ar  VASFSS---------LYQSSCQGKLHRSISWA
SLRIAKPPKQ-------------GLNNSPPVKEFKESSAVDAFRSRSISVSEHVVR  Sh  VASFSS---------MYQSSCQGKLHRSVSWA
SLRIAKPPKQ-------------GLNNSPPVKEFKESSAVDAFRSRSISVSEHVVR  Cu  VTSFSS---------MYQSSCQGKLHKSVSWA
SLRIARPPKQ-------------GLNNSPPVKEFKESSAVDAFRSRSISVSEHVVR  Op  VASFSS---------MYQSSCQGKLHRSVSWA
SLRIAKSVRP-------------GLANSPPVKEIRESPAANAFRCRSISVSEHAVH  On  VASFSS---------MLLSSCQGKLHRSVSWA
SLRLAKNAKQ-------------GLNNSPPVKELKEPSAVDAFRSRSISVSEHVVR  Gg  VASFSS---------MYQSSCQGKLHRSISWA
SLRLAKNAKQ-------------GLNNSPPVKELKEPSAVDAFRSRSISVSEHVVR  Me  VASFSS---------MYQSSCQGKLHRSISWA
SLRLAKNAKQ-------------GLNNSPPVKELKESSAVDAFRSRSISVSEHVVR  Ai  VASFSS---------MYQSSCQGKLHRSISWA
SLRLAKNAKQ-------------GLNNSPPVKELKESSAVDAFRSRSISVSEHVVR  Cl  VASFSS---------MYQSSCQGKLHRSISWA
SLRLAKNAKQ-------------GLNNSPPVKELKESSAVDAFRSRSISVSEHVVR  Ep  VASFSS---------MYQSSCQGKLHRSISWA
SLRLAKNAKQ-------------GLNNSPPVKELKESSAVDAFRSRSISVSEHVVR  Ae  VASFSS---------MYQSSCQGKLHRSISWA
SLRLAKNAKQ-------------GLNNSPPVKELKESSAVDAFRSRSISVSEHVVR  Mu  VASFSS---------MYQSSCQGKLHRSISWA
SLRLAKNAKQ-------------GLNNSPPVKELKEPSAVDAFRSRSISVSEHVVR  Ps  VASFSS---------MYQSSCQGKLHRSISWA
SLRLAKNAKQ-------------GLNNSPPVKELKEPSAVDAFRSRSISVSEHVVR  Uh  VASFSS---------MYQSSCQGKLHRSISWA
SLRIAKNAKQ-------------GLNNSPPVKELKEPSAVDAFRSRSISVSEHVVR  Cm  VASFSS---------MYQSSCQGKLHRSISWA
SLRIAKNAKQ-------------GLNNSPPVKELKEPSAVDAFRSRSISVSEHVVR  As  VASFSS---------MYPSSCQGKLHRSISWA
SLRIAKNAKQ-------------GLNNSPPVKELKEPSAVDAFRSRSISVSEHVVR  Gn  VASFSS---------MYQSSCQGKLHRSISWA
SLRIAKNAKQ-------------GLNNSPPVKELKEPSAVDAFRSRSISVSEHVVR  Ck  VASFSS---------MYQSSCQGKLHRSISWA
SLRIAKNARQ-------------GLNNSPPVKELKEPSAVDAFRSRSISVSEHVVR  Cd  VASFSS---------MYQSSCQGKLHRSISWA
SLRIAKNARQ-------------GLNNSPPVKELKEPSAVDAFRSRSISVSEHVVR  Te  VASFSS---------MYQSSCQGKLHRSISWA
SLRIARNAKQ-------------GLNHSPPVKELKEPSAVDVFRSRSISVSEHVVR  If  VASFSS---------LYQSSCQGKLHRSISWA
SLRIARNAKQ-------------GLNHSPPVKELKETSAVDVFRSRSISVSEHVVR  Tl  VASFSS---------LYQSSCQGKLHRSISWA
SFKVAKSARQ-------------SLNHSPPVKELRETSPAEAFRSRSISVSDHAAH  Ac  VASFSS---------VYLDSSQEKLYRSISWA
NFKVAKNARP-------------NLNHSPPVKALKEPSAVDAFRSRSISVSDHAAH  Pd  VASFSA---------MGLTSSQGRLHRSISWA
NFKVAKNARP-------------NLNPSPSVRALKDPSAVDAFRSRSISVSDHAAH  Tk  VASFSA---------MGLTSSQGGLYRSISWA
NFKVAKNARP-------------NLNQSPPVRALKDPSAVDAFRSRSISVSDHAAH  Oh  VASFSA---------MGLTSSQGGLYRSISWA
NFKVAKNARP-------------NLNQSPPVKALKDPSAVDAFRSRSISVSDHAAH  Cr  VASFSA---------MGLTSFPGGLHRSISWA
NFKVAKNARP-------------NLNQSPPVKAPKDPSAVDAFRSRSISVSDHAAH  Vb  .....L---------MGLTSPPGGLRRSISWA
........................................................  Ug  VASVSL---------SSQSSGPGKLLRSISWA
........................................................  Rx  VASVSP---------ISPSSGQGKLLRSISWA
SLKLSKPSKP-------------GLSDSSPVKEMRDLS--NAFRSRSISVSERAAY  Np  VASVAP---------ILPSSGQGKLLRSISWA
SLKLSKHTKP-------------GFSDTSPVKEMRDLS--NAFRSRSISVSEHVLH  Xt  VASVSL---------VTQSSGQGKLLRSISWA
SLRLSKSAKP-------------GMSSTSPVKEMRDLS-ANAFRSRSISVSEHA..  Hc  VASFSS---------IHP..............
SLRISKNLKL-------------GVSNTSPVKEMKELSSADAFRSRSISVSEHAVR  Sx  ..................SSGQGKLRRSVSWA
SLRISKSVKQ-------------GVNNTSPVKEMKELSSADAFRSRSISVSEHAFR  Cy  VASFSS---------IHPSSGQGMLRRSVSWA
SLRISKSVKQ-------------GVNNTSPVKEMKELSSADAFRSRSISVSEHAFR  Sv  VASFPS---------IHPSSGQGKLRRSVSWA
SLRGSKPQKP-------------GLNSS-QIKDMKDLSAVEAFRSRSISVSEHAVR  Rz  VASFSS---------MCQTSCRGELHRSISWA
SSRTTKSLKP-------------GLNNS-QIKDMKELSAVEAFRSRSISVSEHAAR  Ef  VGSFSS---------NCLTSSRGKLHRSISWA
SLRTAKTLKP-------------GFNSS-PVKEMKELSAVDAFRSRSISVSEHAVR  Li  VASFSS---------MFQSSCQEKLHRSISWA
SLRTARVPKQ-------------GLTSS-PVKELKDLSAMDAFRSRSISVSEHAVR  Lo  VASFSS---------LYLPGSQVKLHRSISWA
SVRTARVARQ-------------SQSANSPVKELKDLSAMDAFRSRSISVSEHAVR  Eh  VGSLCS---------LCPSNQPGRLHRSISWA
SLRTAKVAKQ-------------SQSANSPVKELKDLSAMDAFRSRSISVSEHAVR  Ax  VGSLCS---------LCPADQPGRLHRSISWA
SLRTAKVAKQ-------------GPGSNSPVKELKDLSAMDAFRSRSISVSEHAVR  Gf  VASLSS---------LCLSDTPGRLHRSISWA
SLRTAKVAKQ-------------GPSSNSPVKELKDLSAMDAFRSRSISVSEHAVR  Aj  VASLSS---------LCLSAPPARLYRSISWA
SLRAAKVVKQ-------------GPSSNSPIKELKDLSAMDAFRSRSISVSEHAVR  Qh  ....CV---------LCPSELQGRLTRSISWA
SLRTTKVAKQ-------------GPSANSPVKDLKDLSAMDAFRSRSISVSEHAVR  Gi  VGALSS---------LCSPDSPGRFHRSISWA
SLRTTKVAKQ-------------GPSANSPVKDLKDLSAMDAFRSRSISVSEHAVR  Pp  VGALSS---------LCSPDTPGRFHRSISWA
SLRTTKVAKQ-------------GPSANSPVKDVKDFSAMDAFRSRSISVSEHAVR  Dr  VASLSS---------LCSSESSGRFHRSISWA
SLRAAKMAKAHQQQQQASMAAGVAASTGSPVKELRDLSAMDAFRSRSISVSEHAVR  Ta  VGSLSS-LGLS----FAPPPVPVRLQRSISWA
SLRAAKMAKAHLQQQQASMAAGVAASTGSPVKELRDLSAMDAFRSRSISVSEHAVR  Se  VGSLSS-LGLS----FAPSPVPGRLQRSISWA
SLRAAKMARAH//QQQAATASGVATSSSSPVKE---LSAMDAFRSRSISVSEHAVR  Ex  VDSLSS-MGLSVVPPPALPPAPGRLQRSISWA
SHRAPKVAKA-----------AAGNSSGSPVKELRDRSAMEAFRCRSISVSEHAVR  Ol  VGVNLV---------LLLDRHRKELWDTIPFA
```

```
RSKVARAAAA------------VANSSSSPVKELRDVSAMDAFRSRSISVSEHAVR  Gd  ................................
SLRAAKVAKA--------AAVAVANS-QSPVKELRDLSAMDAFRSRSISVSDHAVR  Pw  VGGSLWSLGQGSV--PSGPPAPGRLHRSISWA
SLRAAKVAKA--------AAVAVANS-QSPVKELRDLSAMDAFRSRSISVSDHAVR  Pg  VGGSLWSLSQGWV--PSGPPAPGRLHRSISWA
SLRAAKVAKA--------AAVAVANS-QSPVKELRDLSAMDAFRSRSISVSDHAVR  Bp  VGGSLWSLGQGSV--PPGPLGPGRLHRSISWA
SLRAAKVAKA--------AAVAVANKSQSPVKELRDLSAMDAFRSRSISVSDHAVR  Sq  VGGSLWSLGQGSV--PSGPQGPGRLHRSISWA
SLRATKVAKA---------AATVANSSSSPVKELRDLSAMDAFRSRSISVSEHAVR  Hi  VGDSLWSLVQGSA--PSGPPPLGRLHRSISWA
SLRAAKVANA---------AAAVANSSGSPVKELRDLSAIDAFRSRSISVSEHAVR  Sl  ................................
SLRAAKMAKA---------AAAVANNSSSPVKELRDLSAMDAFRSRSISVSEHAVR  Vi  VCGSLWSLGLGSA--PSGPPAPGRLHRSISWA
SLRAAKVAKA---------AAAVANSSSSPVKELRDLSAMDAFRSRSISVSEHAVR  Nf  VGSSLWSLGLGSA--PPGPPVPGRLHRSISWA
SLRAAKVAKA---------AAAVANSSSSPVKELRDLSAMDAFRSRSISVSEHAVR  Lr  VGGSLWSLGSGSA--PPGSPAPGRLHRSISWA
SMRAAKVAKA---------AAAVANSSGSPVKELRDLSAMDAFRSRSISVSEHAVR  Ni  VGGSLWSLGSGSG--PPGPPAPGRLHRSISWA
SLRAAKVAKA---------AAAVANSSSSPVKELRDLSAMDAFRSRSISVSEHAVR  Sw  VGGSLWSLGLGTA--PPGPPAPGRLHRSISWA
SLRAAKVAKA---------AAAVANSSSSPVKELRDLSAMDAFRSRSISVSEHAVR  Dl  VGGSLWSLGLGSA--PPGPPAPGRLHRSISWA
SLRAAKVAKA---------AAAVANSSSSPVKELRDLSAMDAFRSRSISVSEHAVR  Er  VGGSLWSLGLGSA--PPGPPAPGRLHRSISWA
...............................KELRDLSAMDAFRSRSISVSEHAVR  Qa  VGGSLWSLGLGSA--PPGPPAPGRLHRSISWA
SLRAAKVAKA---------AAAVANSSSSPVKELRDLSAMDAFRSRSISVSEHAVR  Ab  VGGSLWSLGMGTT--PPGPPAPGRLHRSISWA
SLRAAKVAKA---------AAAVANSSSSPVKELRDLSAMDAFRSRSISVSEHAVR  Ht  VGGSLWSLGLASG--PPGPPAPGRLHRSISWA
SLRAAKVARA---------AAAVANSSSSPVKELRDLSAMDAFRSRSISVSEHAVR  Ow  VGGFLWSQGLGSA--PSGPPAPGRLHRSISWA
SLRAGKVAKP---------AASVANSSSSPVKELRDLSAMEAFRSRSISVSEHAVR  Ff  VGGSLWSLGLASG--PPGPPAPGRLHRSISWA
SLRAGKVAKP---------AASVANSSSSPVKELRDLSAMEAFRSRSISVSEHAVR  Xm  VGGSLWSLGMASG--PPGPPAPGRLHRSISWA
SLRAGKVAKP---------SASVANSSSSPVKELRDLSAMEAFRSRSISVSEHAVR  Cz  VGGSLWSLGMASA--PPAPPAPGRLHRSISWA
SLRAAKVAKA---------AAAVANSSSSPVKELRDLSAMDAFRSRSISVSEHAVR  Ga  VGSALASLGLGSA--PPGLPVPGRLHRSISWA
SLRAAKVAKA---------AAAVANSSSSPVKELRDLSAMDAFRSRSISVSEHAVR  Ot  VGGSLCSLGMGTA--PPGPPAPGRLHRSISWA
SLRAAKVAKA---------AAAVANSSSSPVKELRDLSAMDAFRSRSISVSEHAVR  Hx  VGGSLCSLGMGTA--PPGPPAPGRLHRSISWA
SLRAAKVAKA---------AAAVANSSSSPVKELRDLSAMDAFRSRSISVSEHAVR  Pj  VGGSLCSLGMGTA--PPGPPAPGRLHRSISWA
SLRAAKVAKA---------AAAVANSSSSPVKELRDLSAMDAFRSRSISVSEHAVR  Nb  VGGSLCSLGMGTA--PPGPPAPGRLHRSISWA
SLRAAKVAKA---------AAAVANSSSSPVKELRDLSAMDAFRSRSISVSEHAVR  Mz  VGGSLCSLGMGTA--PAGPPAPGRLHRSISWA
SLRAAKVVKA---------TAAVTNNSSSPVKELRDLSAMDAFRSRSISVSEHAVR  Fr  VGSSLWSLGSGCA--PPGPSAPGRLHRSISWA
SLRAAKVSKA----------AAVANSSSSPVKELRDLSAMDAFRSRSISVSEHANR  Nh  VGGSLWSLGLGCA--PPGPPTPGRLHRSISWA
SLLAAKVSKA----------APVANNSSSPVKELRDLSAMDAFRSRSISVSEHAVR  Ul  VGGSLWSLGLGSG--PPGPSTPGRLHRSISWA
SLRAAKVVKA---------TAAVANNSSSPAKELRDLSAMDAFRSRSISVSEHAVR  Tn  VGSSLWALGSGSA--PSGPPAPGRLHRSISWA
SLRSARPSRQ------------PGLGSSSPIKDIRELPLAVSFRSRSISVSDQVHR  Lc  VTSLICSTVSGDC-SPWQSSCKNKLSRSISWA
SLRSARPSRQ------------PGLGSSSPIKDVRELPLAASFRSRSISVSDQVHR  Lp  VTSLICSTVSGDC-SPWQSSCKNKLSRSISWA
```

Supp. Figure S2. Simple mathematical analysis to compare the relative degree of amino acid conservation in *TSC2* exons 25 and 31 with other *TSC2* exons. A simple mathematical analysis was performed for each amino acid (Aa) position from exon 1 to exon 41. First, the occurrence of each of the 20 amino acids at every Aa position was listed (e.g. serine occurs 153 times in the first Aa position in exon 25, asparagine occurs 4 times in some species in this same Aa position, lysine occurs once and arginine occurs once). The Aa with the highest occurrence was taken (i.e. 153 of serine) and this number was divided by the number of sequences known at that position (i.e. 159 for the four different amino acids). This gives the "conservation ratio" and this was performed for each Aa position. A perfect phylogenetic conservation gives a value of 1 and the presence of other Aa residues at a given position will decrease this value proportionally, whatever the type of Aa substitution. For each exon, an average for all the "conservation ratios" at each Aa residue was calculated. Next, the "average conservation ratios" for all the 41 exons were taken and a "general conservation average" was calculated. For each Aa position, this "general conservation average" was subtracted from the "conservation ratio" of that Aa position to give a "relative conservation index" which was used to plot the graph.

Graphs showing the relative conservation index at each amino acid residue. Zero on the Y-axis corresponds to the "general conservation average" calculated from the "average conservation ratios" for exons 1-41. Values above zero indicate more conservation than average and values below zero mean more variation than the average. **(A)** Exon 25, **(B)** exon 31, **(C)** exon 7 and **(D)** exon 22. Exons 7 and 22 are examples of exons that are comparatively more conserved than exons 25 and 31.

Supp. Figure S2

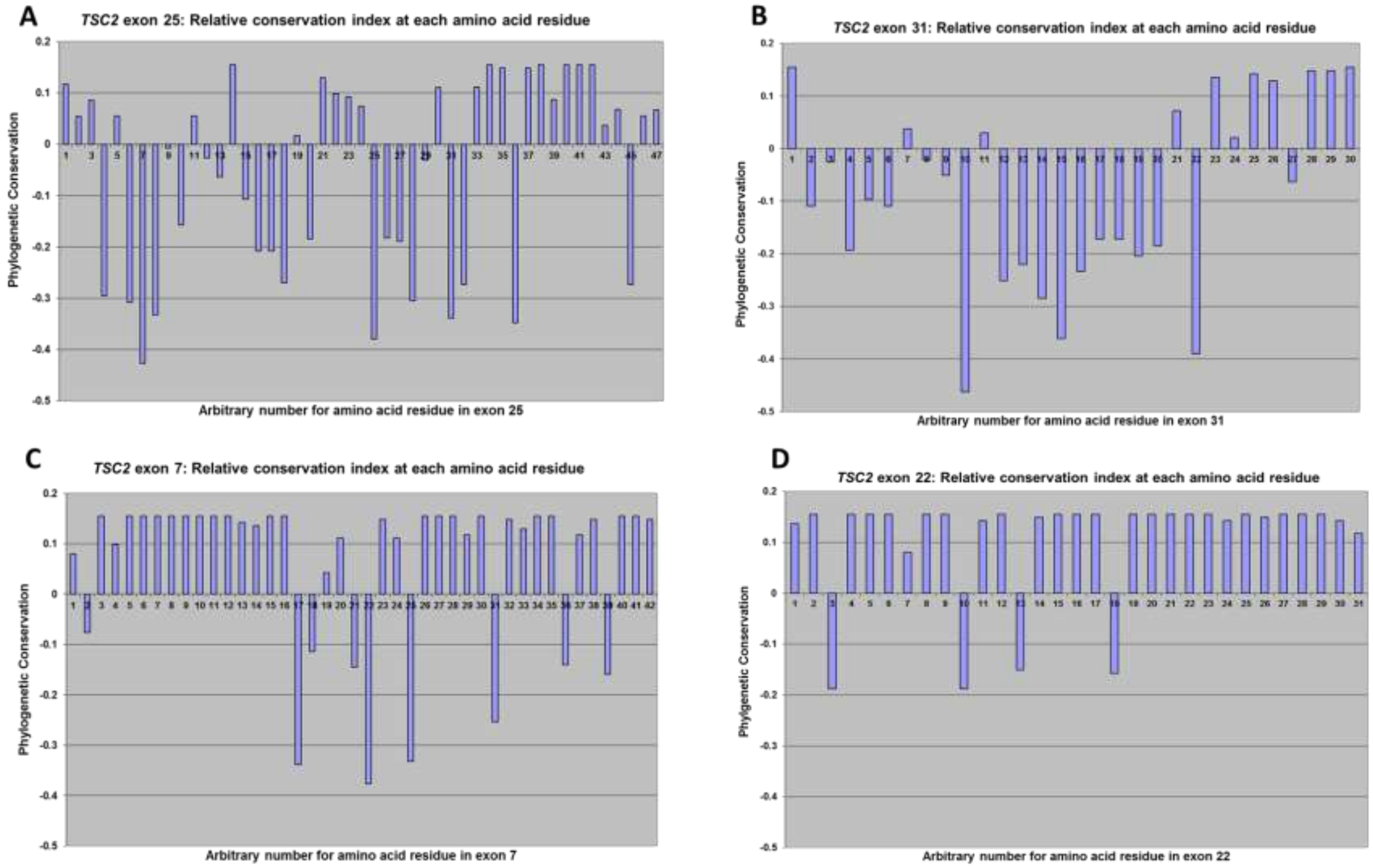

Supp. Figure S3. Application of the Scorecons algorithm to determine the degree of amino acid conservation in *TSC2* exons 25 and 31 compared to other *TSC2* exons. To examine conservation across the TSC2 protein, two versions of the multi-species amino acid alignment were prepared, one with and the other without fish sequences. The conservation score for each amino acid position in the multiple sequence alignment, from exons 1-41, was calculated using the Scorecons algorithm [Valdar, 2002]. These conservation scores were extracted for each exon and plotted (Supp. Figure S3, A and B). Using the box plot (Supp. Figure S3, A and B) as a guide, two of the exons showing high conservation score distributions were chosen, as well as exons 25 and 31, and line plots drawn to show the conservation score at each alignment position within the exon (Supp. Figure S3, C and D).

Supp. Figure S3A-B. The boxplots show conservation across all *TSC2* exons. Each box represents an exon. The black bar within each box is the median score among the scores of all amino acids within that exon. The top and lower half of each box represents 25% of scores above the median and 25% of scores below the median, respectively; the entire box has 50% of all scores. Lines (whiskers) above and below each box represent scores that are outside 50% of the data, i.e. these are 25% of scores above the 75% percentile and 25% below the 25% percentile respectively. Black dots are the outliers. **(A)** The alignment used had fish sequences and no gaps. **(B)** Fish sequences and gaps were removed from the alignment.

Supp. Figure S3C-D. The line plots show the conservation score at each amino acid residue in the alignment. Conservation scores above 0.7 are considered significant. ‘Troughs’ in the line plots (close to zero) are due to amino acids that are present in only a few species. Exons 5, 7 and 22 represent exons where the distribution of conservation scores was higher than in exons 25 and 31. **(C)** The alignment used in the Scorecons algorithm had fish sequences but no gaps. **(D)** Fish sequences and gaps were removed from the alignment prior to running the Scorecons algorithm.

Supp. Figure S3A-B

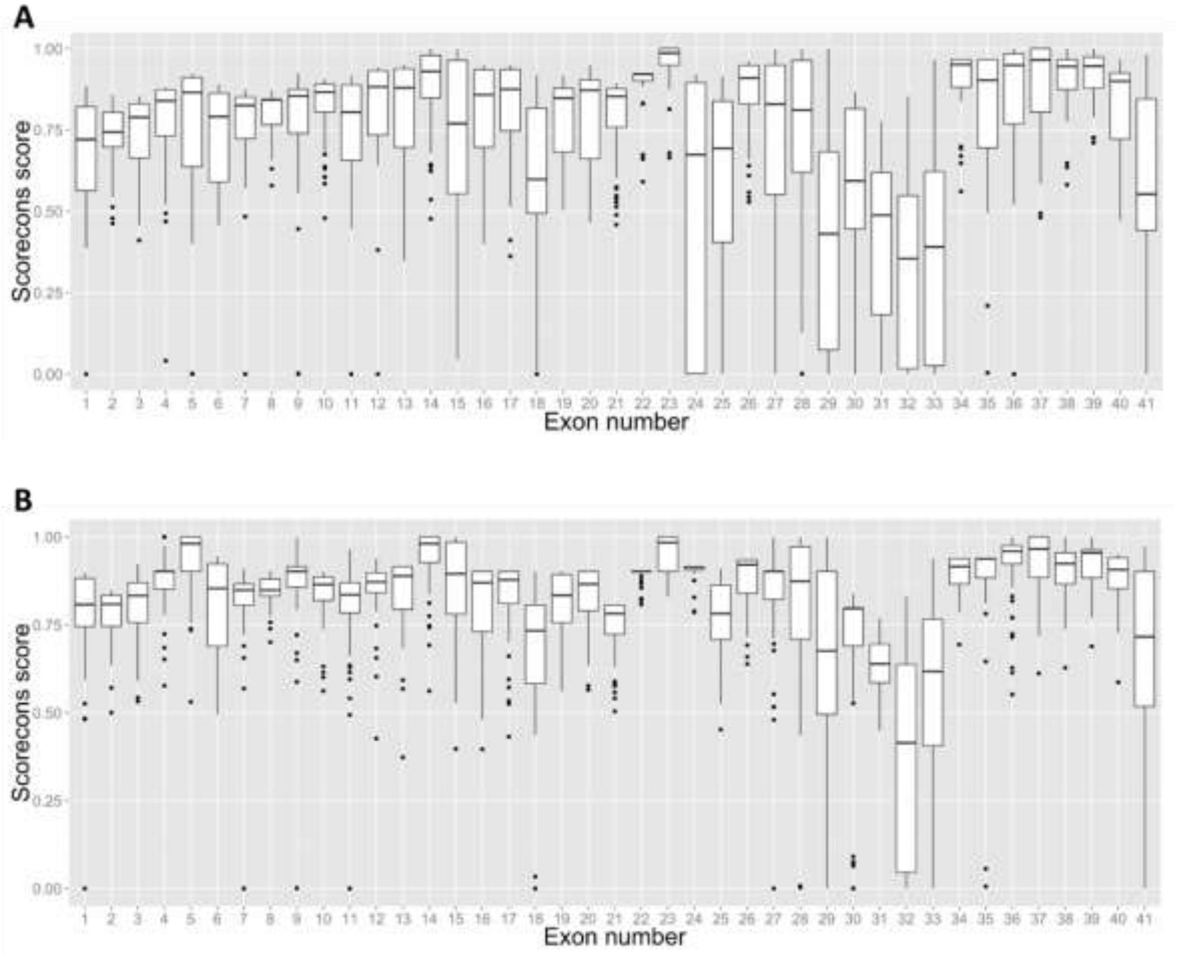

Supp. Figure S3C

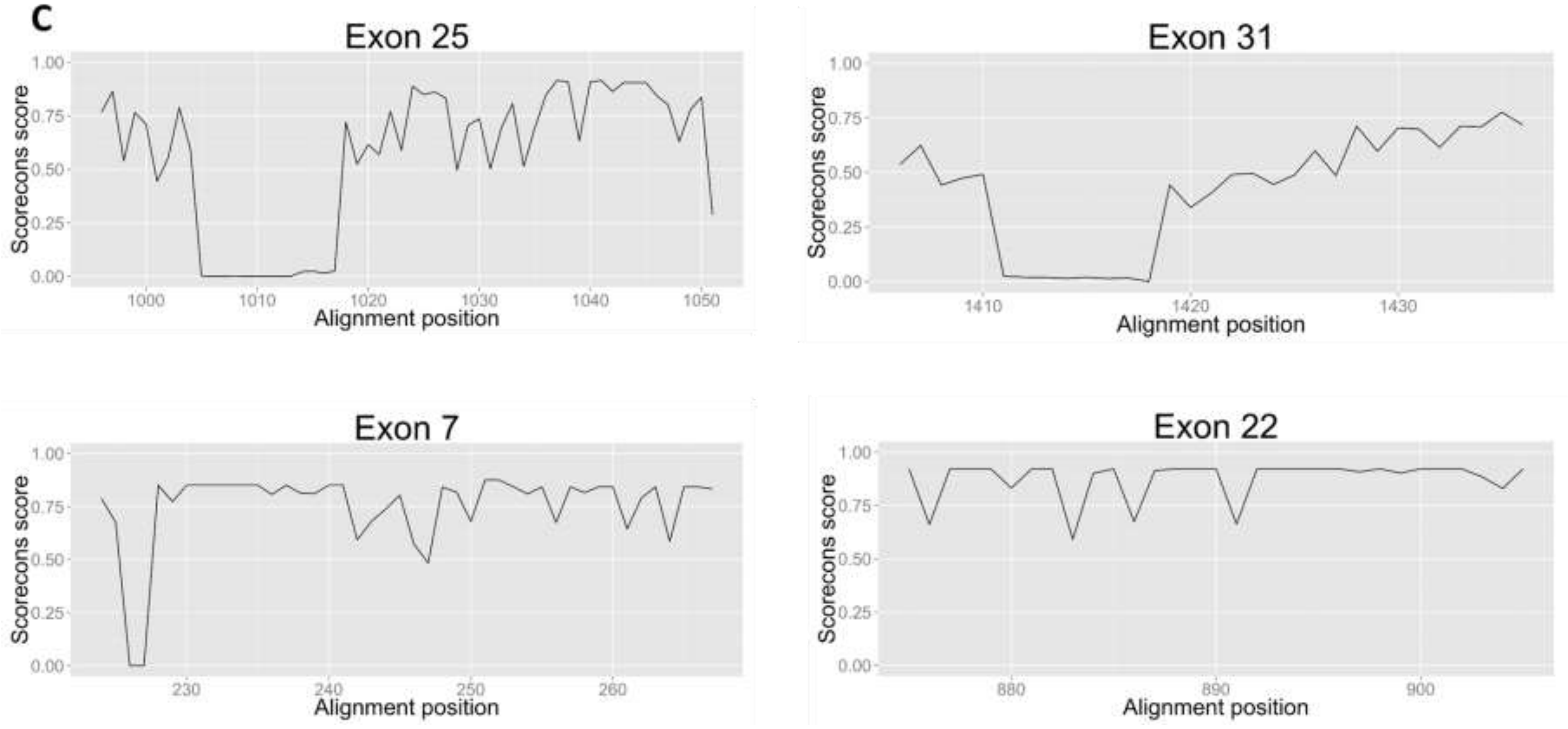

Supp. Figure S3D

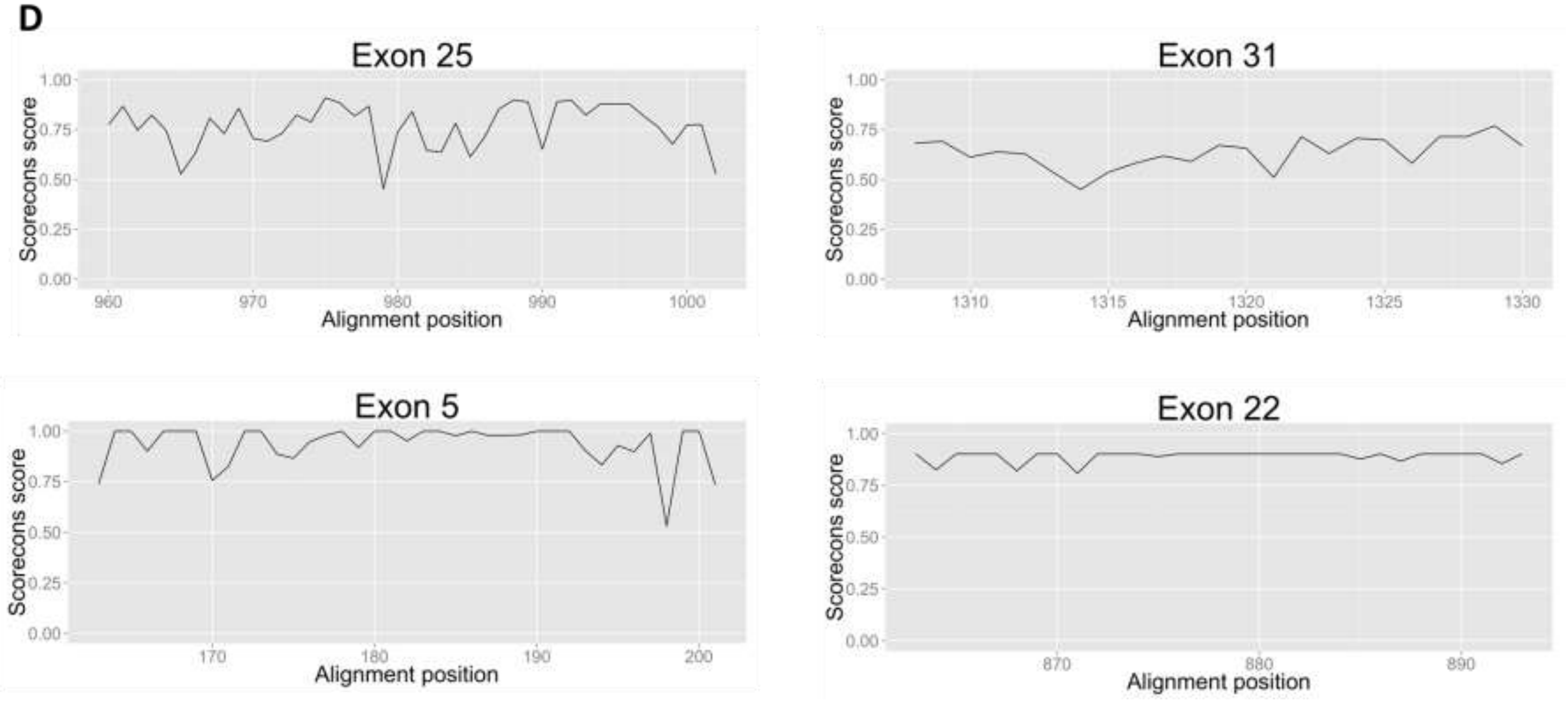

Supp. Figure S4. Boxplots of inclusion ratios of exon 25 and exon 31 in junction reads in *TSC2* RNA-seq data from the Genotype-Tissue Expression (GTEx) project.

For each tissue, ratios indicating the inclusion of exon 25 in the 3’ splice site of the upstream exon 24 were extracted for each sample and used to generate associated boxplots (Supp. Figure S4, A) (see the legend for Supp. Table S3 for details on generating the ratios). A separate graph of boxplots was generated for ratios denoting the inclusion of exon 25 in the 5’ splice site of the downstream exon 26 (Supp. Figure S4, B). Similarly, two separate graphs of boxplots were generated for ratios showing the inclusion of exon 31 in the 3’ splice site of the upstream exon 30 and inclusion in the 5’ splice site of the downstream exon 32 (Supp. Figure S4, C-D). Data from the two cell lines (EBV-transformed lymphoblastoid cell lines and cultured fibroblasts) in the collection were excluded. All boxplots generated (Supp. Figure S4, A-D) were based on sample tissue type (retrieved from the variable named as “SMTSD” and described as “Tissue Type, more specific detail of tissue type”).

Boxplots of ratios showing the inclusion of exon 25 in the overall junction reads of its adjacent **(A)** upstream exon 24 and **(B)** downstream exon 26; inclusion ratios for exon 31 in the overall junction reads of its adjacent **(C)** upstream exon 30 and **(D)** downstream exon 32. The ratios give an indication of the splicing in of exon 25 or exon 31. Black bar within each box is the median value and the black dots are the outliers. Analysis was based on the longest *TSC2* transcript, cDNA sequence accession number (GenBank NM_000548.3, GI:116256351).

Supp. Figure S4A

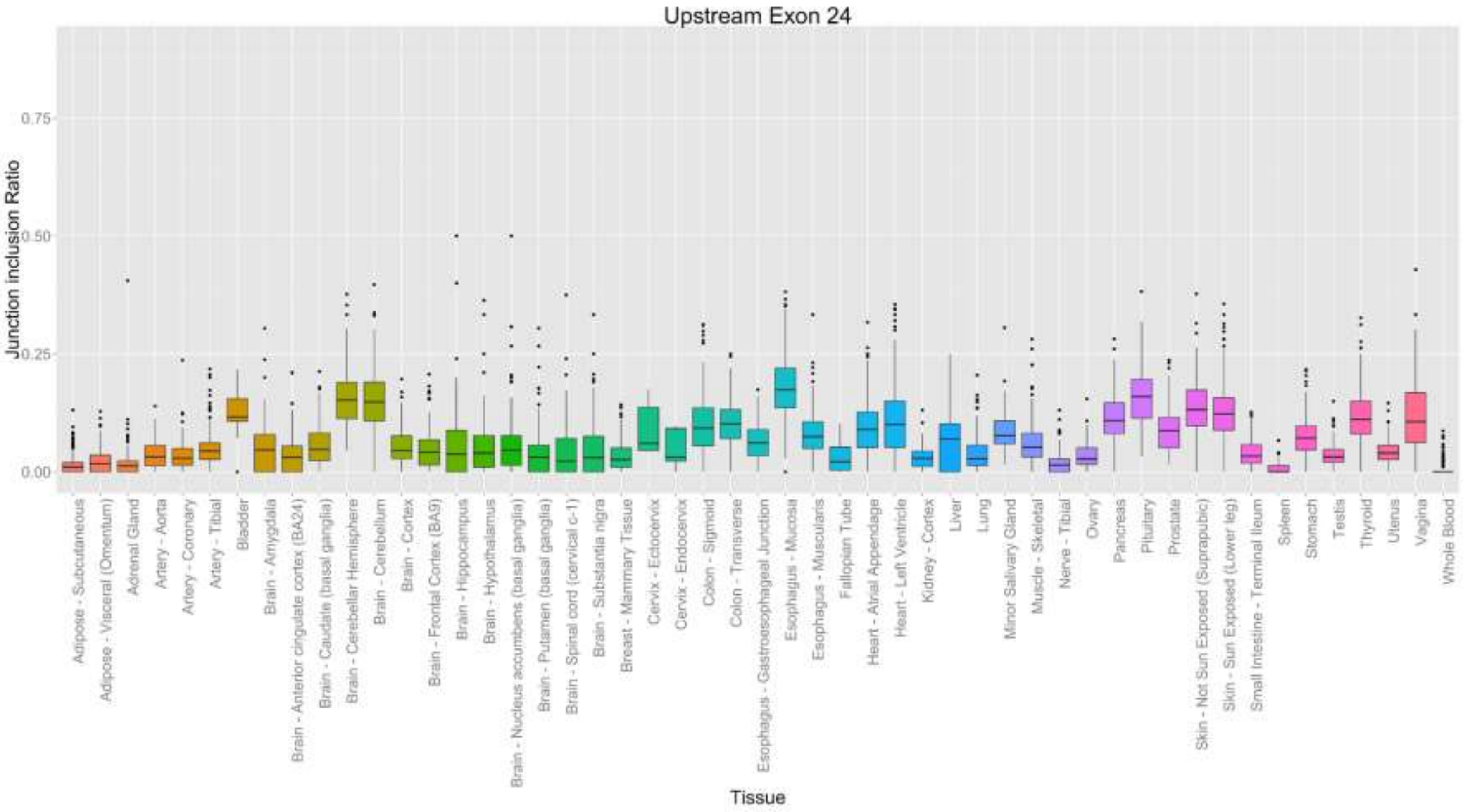

Supp. Figure S4B

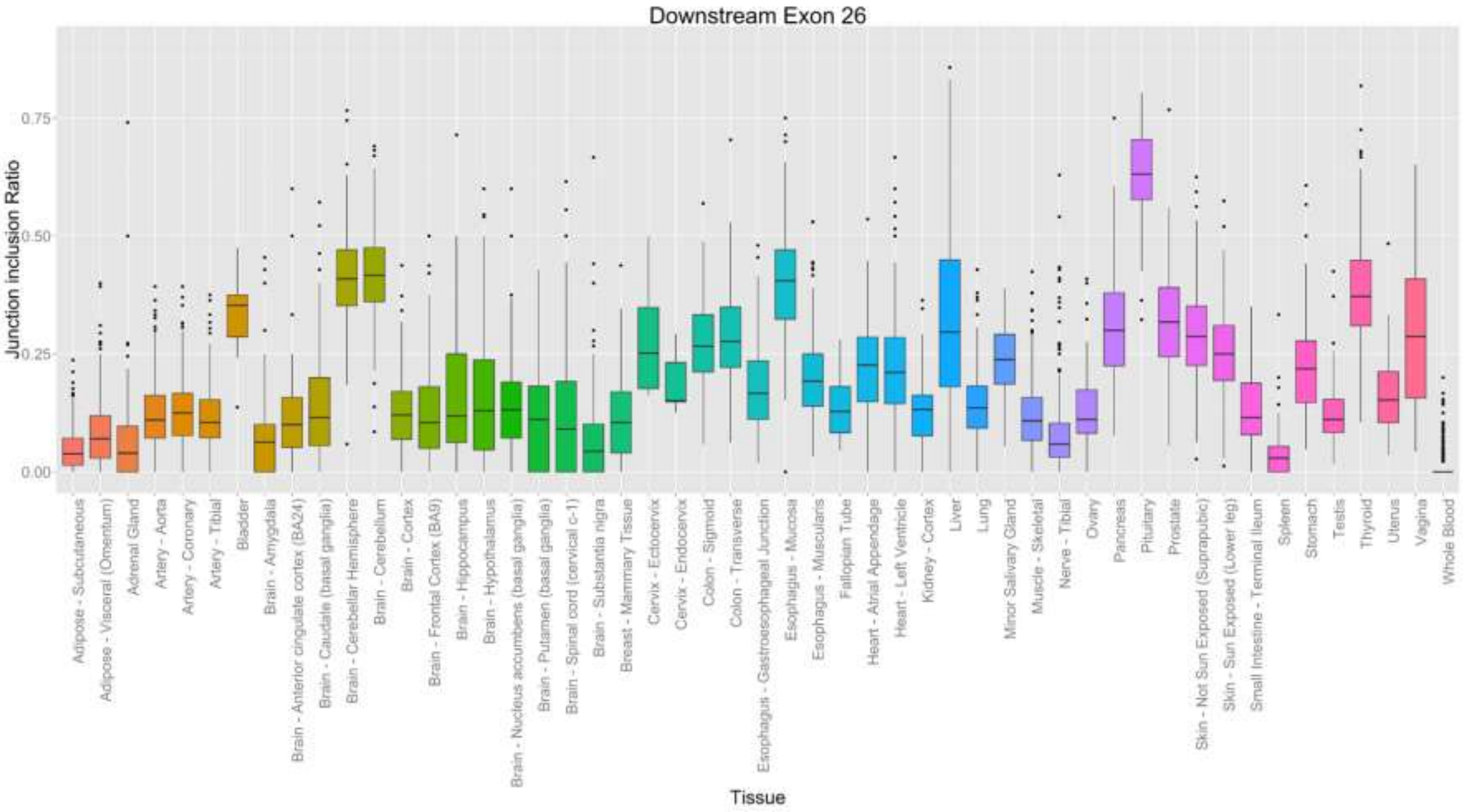

Supp. Figure S4C

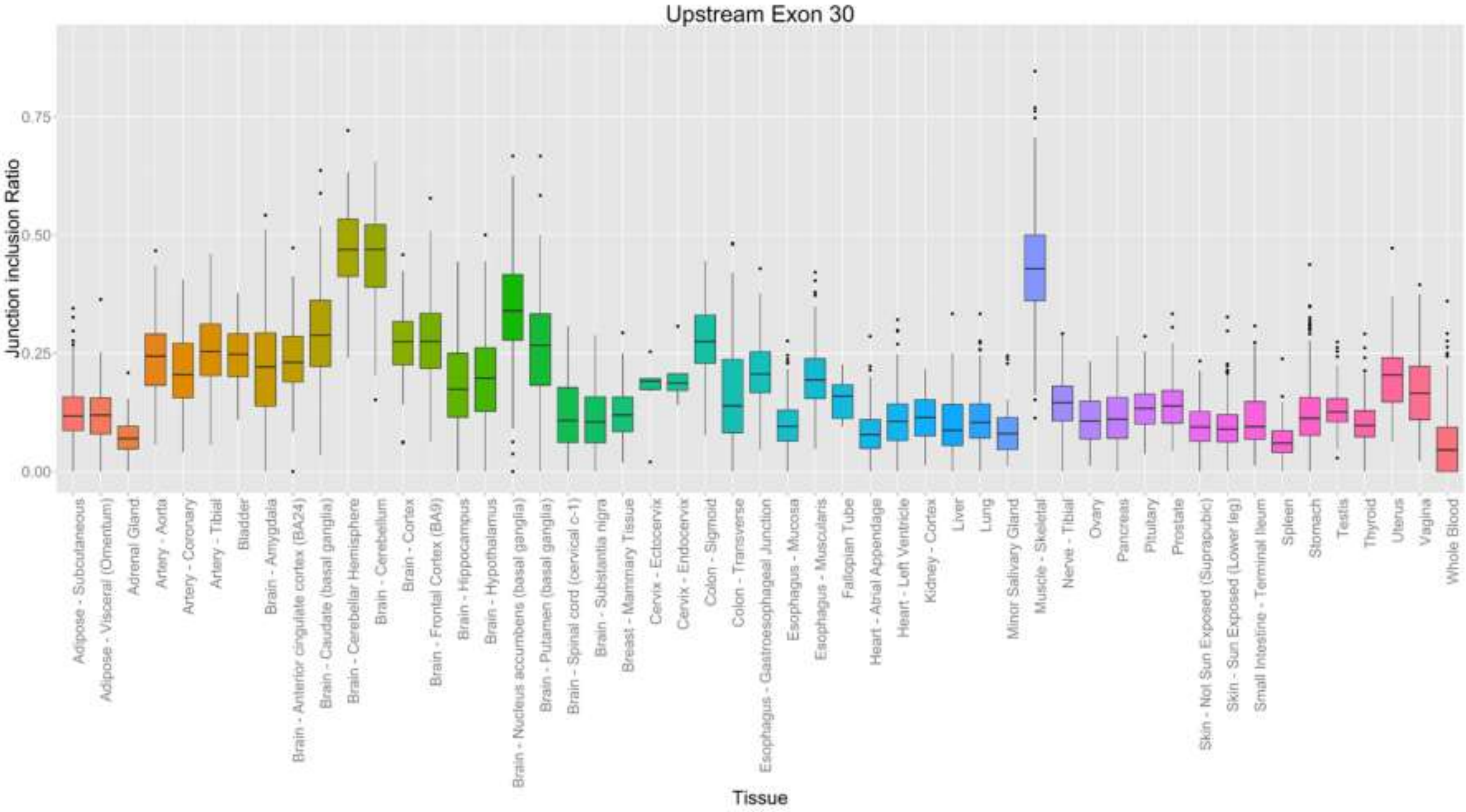

Supp. Figure S4D

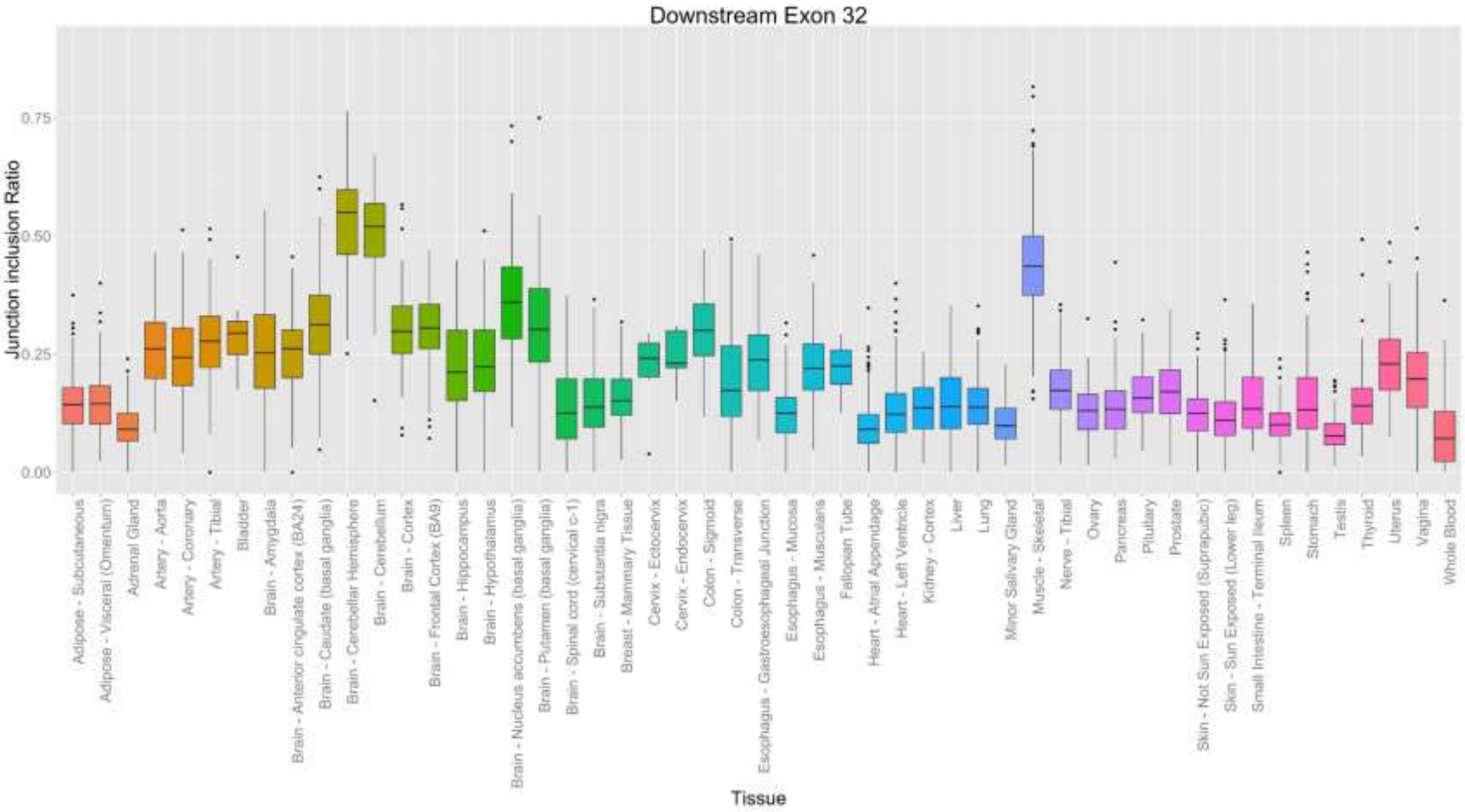

Supp. Figure S5. Differences in expression of *TSC2* exons 25 and/or 31 in different adult tissues. Screenshot of intron spanning tracks from some BodyMap2 tissues showing the inclusion of exons 25 and/or 31 in TSC2 transcripts. Shallow wells indicate the inclusion of exon 25 or 31. NCBI *Homo sapiens* annotation release107; https://goo.gl/S3jztc (accessed November 2015).

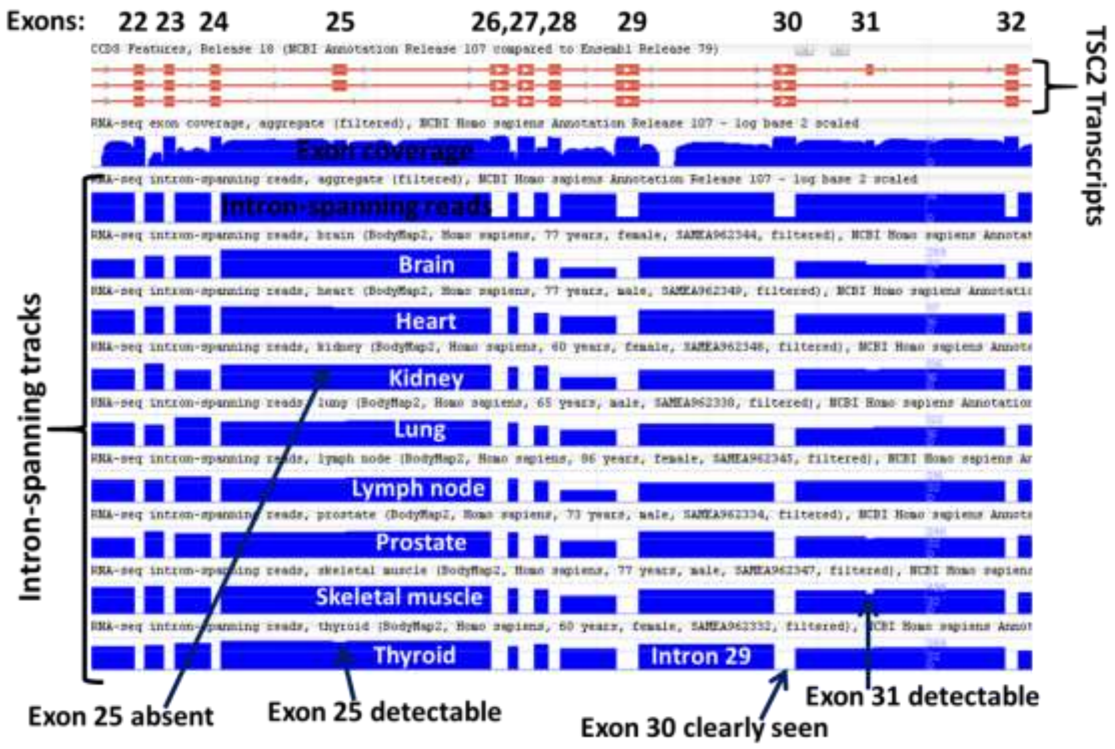

Supp. Table S1. Statistical analysis using Wilcoxon Rank-Sum tests

(A)

**Exons compared in test**					**Exons compared in test**			
**First**	**Second**	**P-value**	**Is significant**		**First**	**Second**	**P-value**	**Is significant**
25	1	0.25072			31	1	5.02E-006	*
25	2	0.1028142			31	2	1.80E-007	*
25	3	0.04757836	*		31	3	1.95E-008	*
25	4	0.000291284	*		31	4	6.65E-011	*
25	5	0.000631023	*		31	5	2.82E-006	*
25	6	0.07072193			31	6	5.88E-005	*
25	7	0.003755296	*		31	7	4.74E-009	*
25	8	0.002860204	*		31	8	3.12E-009	*
25	9	6.89E-005	*		31	9	4.37E-009	*
25	10	3.66E-006	*		31	10	1.08E-011	*
25	11	0.006044311	*		31	11	8.25E-008	*
25	12	4.60E-006	*		31	12	3.95E-009	*
25	13	0.000104771	*		31	13	4.68E-008	*
25	14	9.15E-011	*		31	14	8.57E-012	*
25	15	0.002784863	*		31	15	2.55E-006	*
25	16	2.43E-005	*		31	16	7.00E-009	*
25	17	8.91E-006	*		31	17	7.14E-009	*
25	18	0.4851082			31	18	0.00092201	*
25	19	0.000181844	*		31	19	1.25E-009	*
25	20	4.56E-005	*		31	20	6.45E-010	*
25	21	3.58E-005	*		31	21	2.57E-011	*
25	22	3.87E-010	*		31	22	7.00E-011	*
25	23	3.05E-013	*		31	23	6.12E-012	*
25	24	0.3862131			31	24	0.2175739	

Exons compared in test					Exons compared in test			
**First**	**Second**	**P-value**	**Is significant**		**First**	**Second**	**P-value**	**Is significant**
25	26	3.36E-010	*		31	25	4.69E-003	*
25	27	0.001971423	*		31	26	3.00E-012	*
25	28	0.00049156	*		31	27	3.20E-005	*
25	29	0.9926883			31	28	7.18E-007	*
25	30	0.7862612			31	29	0.7070871	
25	31	0.995428			31	30	0.00523674	*
25	32	0.9999548			31	32	0.9817215	
25	33	0.9999638			31	33	0.8567492	
25	34	6.82E-008	*		31	34	4.37E-009	*
25	35	3.26E-005	*		31	35	9.46E-008	*
25	36	1.74E-010	*		31	36	1.59E-011	*
25	37	2.74E-010	*		31	37	3.32E-012	*
25	38	7.39E-009	*		31	38	4.94E-010	*
25	39	9.43E-010	*		31	39	2.61E-011	*
25	40	1.31E-006	*		31	40	1.98E-009	*
25	41	0.5666149			31	41	0.02112048	*

(B)

Exons compared					Exons compared			
**First**	**Second**	**P-value**	**Is significant**		**First**	**Second**	**P-value**	**Is significant**
25	1	0.1532412			31	1	1.54E-06	*
25	2	0.5114529			31	2	6.83E-07	*
25	3	0.05071851			31	3	1.37E-07	*
25	4	1.94E-07	*		31	4	6.24E-11	*

**Exons compared**					**Exons compared**			
**First**	**Second**	**P-value**	**Is significant**		**First**	**Second**	**P-value**	**Is significant**
25	5	2.31E-10	*		31	5	1.97E-10	*
25	6	0.06776754			31	6	0.000176959	*
25	7	0.04054813			31	7	1.20E-08	*
25	8	0.003783145	*		31	8	3.03E-09	*
25	9	1.56E-06	*		31	9	2.10E-08	*
25	10	0.00037038	*		31	10	1.08E-09	*
25	11	0.06593359			31	11	1.65E-06	*
25	12	0.000187739	*		31	12	2.14E-08	*
25	13	0.00026819	*		31	13	5.62E-07	*
25	14	5.18E-12	*		31	14	2.14E-11	*
25	15	0.00047942	*		31	15	1.53E-06	*
25	16	0.01774143	*		31	16	3.77E-06	*
25	17	0.002512198	*		31	17	1.74E-06	*
25	18	0.9958998			31	18	0.03977984	*
25	19	0.02337641	*		31	19	1.28E-07	*
25	20	0.002569909	*		31	20	2.93E-08	*
25	21	0.9683016			31	21	6.06E-08	*
25	22	2.85E-10	*		31	22	3.50E-11	*
25	23	6.52E-12	*		31	23	6.36E-11	*
25	24	1.11E-11	*		31	24	3.67E-11	*
25	26	2.07E-08	*		31	25	2.37E-06	*
25	27	1.81E-06	*		31	26	9.58E-12	*
25	28	0.02291789	*		31	27	2.14E-08	*
25	29	0.9729662			31	28	1.49E-05	*
25	30	0.9701119			31	29	0.2791705	
25	31	0.9999978			31	30	9.35E-06	*
25	32	1			31	32	0.9998668	
25	33	1			31	33	0.7540439	

**Exons compared**					**Exons compared**			
**First**	**Second**	**P-value**	**Is significant**		**First**	**Second**	**P-value**	**Is significant**
25	34	1.51E-07	*		31	34	2.82E-09	*
25	35	2.42E-07	*		31	35	4.90E-08	*
25	36	4.21E-12	*		31	36	2.02E-11	*
25	37	5.70E-11	*		31	37	2.07E-11	*
25	38	5.92E-07	*		31	38	4.03E-09	*
25	39	4.27E-09	*		31	39	5.75E-10	*
25	40	3.14E-07	*		31	40	8.42E-10	*
25	41	0.8774469			31	41	0.08844522	

To observe whether the amino acid conservation is reduced in exons 25 and 31, two-sample Wilcoxon Rank-Sum tests were performed in R [R Core Team, 2015]. Pairwise comparisons of the distribution of conservation scores (from the Scorecons output; Supp. Figure S3) in exons 25 and 31 against the distribution of scores in all other exons were performed. The alternative hypothesis, that assessed whether the distribution of conservation scores in either exon 25 or 31 consisted of lower scores than the distribution of scores in the exon being compared against, was accepted if the P-value was less than 0.05.

P-values for **(A)** the alignment containing fish sequences and no gaps, **(B)** the alignment without fish sequences and without gaps.

* = The distribution of conservation scores in exon 25 and exon 31 is lower than that in the exon it is being compared against (P <0.05).

Supp. Table S2. Junctions used in the analysis of *TSC2* exon 25 and exon 31 inclusion in RNA-seq data from the Genotype-Tissue Expression (GTEx) project

(A)

**Exon 24-25 junction**	**Exon 25-26 junction**
Chr16 2131799 - 2132437<br><br>Chr16 2131799 - 2133696	Chr16 2131799 - 2133696<br><br>Chr16 2132505 - 2133696<br><br>Chr16 2133255 - 2133696

(B)

**Exon 30-31 junction**	**Exon 31-32 junction**
Chr16 2131799 - 2132437<br><br>Chr16 2131799 - 2133696	Chr16 2131799 - 2133696<br><br>Chr16 2132505 - 2133696

Junctions for **(A)** *TSC2* exon 25 and **(B)** *TSC2* exon 31. Locations on human chromosome 16 are based on the human GRCh37/hg19 assembly (GenBank NC_000016.9, GI:224589807).

Supp. Table S3. Bioinformatic analysis of exon 25 and 31 junction reads in *TSC2* RNA-seq data from the Genotype-Tissue Expression (GTEx) project

Tissues	Upstream Exon 24	Downstream Exon 26	Upstream Exon 30	Downstream Exon 32
**Adipose - Subcutaneous**	0.01409105	0.04792035	0.12291388	0.14565593
**Adipose - Visceral (Omentum)**	0.022672777	0.082869419	0.120815506	0.146664762
**Adrenal Gland**	0.020225921	0.066287072	0.07408006	0.095487213
**Artery - Aorta**	0.035282406	0.121793609	0.240354412	0.2659411
**Artery - Coronary**	0.035662563	0.130931723	0.213727638	0.245063302
**Artery - Tibial**	0.048173881	0.114431846	0.254826138	0.277430211
**Bladder**	0.123628841	0.331201704	0.243729757	0.289355936
**Brain - Amygdala**	0.054047022	0.080329628	0.229699011	0.257399634
**Brain - Anterior cingulate cortex (BA24)**	0.041561996	0.112124975	0.237734988	0.255202537
**Brain - Caudate (basal ganglia)**	0.056071584	0.13563801	0.291088853	0.315305775
**Brain - Cerebellar Hemisphere**	0.158887149	0.416086975	0.470370299	0.524732279
**Brain - Cerebellum**	0.15395163	0.414849016	0.453655454	0.507294428
**Brain - Cortex**	0.054630724	0.130267984	0.269260165	0.298300882
**Brain - Frontal Cortex (BA9)**	0.04947483	0.126053018	0.276562073	0.301462928
**Brain - Hippocampus**	0.059477469	0.158323448	0.186482627	0.221682752
**Brain - Hypothalamus**	0.05585973	0.165987625	0.205489816	0.233946419
**Brain - Nucleus accumbens (basal ganglia)**	0.059802885	0.143881169	0.332439987	0.358680668
**Brain - Putamen (basal ganglia)**	0.045366073	0.116145572	0.262860362	0.31244466
**Brain - Spinal cord (cervical c-1)**	0.04946101	0.127564764	0.117957873	0.140921309
**Brain - Substantia nigra**	0.05016135	0.084099702	0.114601713	0.151834343
**Breast - Mammary Tissue**	0.03430065	0.110226504	0.123454022	0.159355704

Tissues	Upstream Exon 24	Downstream Exon 26	Upstream Exon 30	Downstream Exon 32
**Cervix - Ectocervix**	0.090393544	0.282877482	0.17019052	0.214625917
**Cervix - Endocervix**	0.048560759	0.189731706	0.202404593	0.242246415
**Colon - Sigmoid**	0.100924745	0.273938328	0.278072191	0.302583365
**Colon - Transverse**	0.102084731	0.286868103	0.164103849	0.193844643
**Esophagus - Gastroesophageal Junction**	0.063694252	0.177721366	0.209604978	0.236559055
**Esophagus - Mucosa**	0.182496634	0.401733246	0.100371876	0.12468536
**Esophagus - Muscularis**	0.080509261	0.200333482	0.198756864	0.223052304
**Fallopian Tube**	0.034292766	0.141372269	0.154480125	0.218404024
**Heart - Atrial Appendage**	0.093938699	0.217557589	0.081684445	0.097090984
**Heart - Left Ventricle**	0.108022517	0.219878249	0.11019094	0.132410012
**Kidney - Cortex**	0.033492331	0.140884779	0.11496433	0.13852617
**Liver**	0.071524724	0.328372113	0.100969789	0.150913351
**Lung**	0.037502464	0.141160506	0.111336462	0.140565326
**Minor Salivary Gland**	0.086621288	0.238872973	0.086548955	0.10805308
**Muscle - Skeletal**	0.059881419	0.118985435	0.428198579	0.436932193
**Nerve - Tibial**	0.018605098	0.085678498	0.145663359	0.175937757
**Ovary**	0.034189097	0.133571912	0.109369286	0.131508968
**Pancreas**	0.111716684	0.305895877	0.115373394	0.140636136
**Pituitary**	0.159719706	0.630210009	0.135221681	0.163651644
**Prostate**	0.092842796	0.317405995	0.139678482	0.178698852
**Skin - Not Sun Exposed (Suprapubic)**	0.136380361	0.289996892	0.096294739	0.12380428
**Skin - Sun Exposed (Lower leg)**	0.127209473	0.253422387	0.095323074	0.116008532

Tissues	Upstream Exon 24	Downstream Exon 26	Upstream Exon 30	Downstream Exon 32
**Small Intestine - Terminal Ileum**	0.041978712	0.135843008	0.113713453	0.152467979
**Spleen**	0.007054071	0.039746212	0.065380441	0.102130347
**Stomach**	0.075144867	0.221847633	0.131974661	0.154362672
**Testis**	0.03538017	0.12261098	0.131682009	0.082889002
**Thyroid**	0.118885011	0.379147623	0.103645345	0.147537852
**Uterus**	0.044822615	0.165290937	0.20509449	0.236319405
**Vagina**	0.123213245	0.292084579	0.16829054	0.20687677
**Whole Blood**	0.002793326	0.009796676	0.060135559	0.082067261

The GTEx project contains samples from 237 postmortem donors [GTEx Consortium, 2015] but only samples from 175 donors were used for RNA sequencing. To assess the inclusion of *TSC2* exons 25 and 31, the junction read counts file was downloaded from GTEx Analysis V6 (dbGaP Accession phs000424.v6.p1). All junctions (Supp. Table S2) connecting exon 25 to other canonical exons were extracted for further analysis. A ratio was taken from the 3' splice site of the upstream exon (exon 24). A ratio was calculated to determine the percentage of exon 24-25 junctions compared to all other junctions originating from the 3' splice site of exon 24. Similarly, the junction between exon 25 and the downstream exon 26 was compared to all junctions originating from the 5' splice site of the downstream exon 26. These results, for each sample, were used to generate associated boxplots (Supp. Figure S4). To obtain a single ratio for each junction in a tissue type, all sample ratios of the same tissue type were averaged (Supp. Table S3).

A similar approach was undertaken for exon 31 (junction details in Supp. Table S2). However, a single junction originating from the 5' splice site of the downstream exon 32 was removed. This appears to be exclusively used by a short transcript (GENCODE id ENST00000569930.1) which has 10 exons starting from exon 32 to 41.

Python v2.7.5 [Python Software Foundation, 2015] and R version 3.1.2 [R Core Team, 2014] was used along with packages ggplot2 [Wickham, 2009], dplyr [Wickham and Francois, 2015] and tidyr [Wickham, 2014] to parse the data and generate the plots (Supp. Figure S4).

The table shows the inclusion ratios for exons 25 and 31 in the junction reads of their respective upstream and downstream exons. Data from two cell lines (EBV-transformed lymphoblastoid cell lines and cultured fibroblasts) were excluded.

Supp. Table S4. Truncating variants in *TSC2* exon 31 reported in EVS and ExAC

***TSC2* exon**	**rs IDs**	**DNA variant**	**Protein variant**	**DNA Reported as**	**Population**	**Minor Allele Frequency**	**Database**
31	Not available	c.3845dup	p.(S1282Rfs*40)	c.3844_3845insG	African American	4/4260 alleles (0.0938%)	EVS
31	Not available	c.3850C>T	p.(Q1284*)		Latino	4/11544 alleles (0.0346%)	ExAC
31	Not available	c.3855delA	p.(Q1286Sfs*39)	c.3855del1	African American	4/4260 alleles (0.0938%)	EVS

Variants reported in EVS (http://evs.gs.washington.edu/EVS/) and ExAC (http://exac.broadinstitute.org/). Nucleotide numbering corresponds to the *TSC2* cDNA sequence (GenBank NM_000548.3, GI:116256351) with +1 as A of the ATG translation initiation codon in the reference sequence, and the initiation codon as codon 1. Variants are described as according to guidelines (http://www.hgvs.org/mutnomen/).

Supp. References

Python Software Foundation. 2015. Python Language Reference, version 2.7. http://www.python.org

R Core Team. 2014. R: A language and environment for statistical computing. R Foundation for Statistical Computing, Vienna, Austria. URL https://www.R-project.org/.

Wickham H. 2009. ggplot2: Elegant graphics for data analysis. Springer New York.

Wickham H. 2014. tidyr: Easily Tidy Data with spread() and gather() Functions. R package version 0.2.0. http://CRAN.R-project.org/package=tidyr

Wickham H. and Francois R. 2015. dplyr: A Grammar of Data Manipulation. R package version 0.4.1. http://CRAN.R-project.org/package=dplyr
